# Classification of Nonenzymatic Homologues of Protein Kinases

**DOI:** 10.1155/2009/365637

**Published:** 2009-09-28

**Authors:** K. Anamika, K. R. Abhinandan, K. Deshmukh, N. Srinivasan

**Affiliations:** ^1^Molecular Biophysics Unit, Indian Institute of Science, Bangalore 560 012, India; ^2^NIBR Biologics Center/Protein Production and Antibodies, Novartis Institutes for BioMedical Research, 4056 Basel, Switzerland; ^3^Department of Developmental Biology, Washington University School of Medicine, 660 South Euclid Avenue, Campus Box 8103, St. Louis, MO 63110, USA

## Abstract

Protein Kinase-Like Non-kinases (PKLNKs), which are closely related to protein kinases, lack the crucial catalytic aspartate in the catalytic loop, and hence cannot function as protein kinase, have been analysed. Using various sensitive sequence analysis methods, we have recognized 82 PKLNKs from four higher eukaryotic organisms, namely, *Homo sapiens*, *Mus musculus*, *Rattus norvegicus*, and *Drosophila melanogaster*. On the basis of their domain combination and function, PKLNKs have been classified mainly into four categories: (1) Ligand binding PKLNKs, (2) PKLNKs with extracellular protein-protein interaction domain, (3) PKLNKs involved in dimerization, and (4) PKLNKs with cytoplasmic protein-protein interaction module. While members of the first two classes of PKLNKs have transmembrane domain tethered to the PKLNK domain, members of the other two classes of PKLNKs are cytoplasmic in nature. The current classification scheme hopes to provide a convenient framework to classify the PKLNKs from other eukaryotes which would be helpful in deciphering their roles in cellular processes.

## 1. Introduction

It is now well known that enzymes, in their role as biocatalysts, are the most important control points in the living organisms, and the catalytic residues of an enzyme are key to its molecular function. Bartlett and colleagues [1] have described pairs of active and inactive enzyme homologues having same structural scaffold but different functions. Catalytically inactive enzyme homologues are represented in a large variety of enzyme families with families of signaling enzymes having high number of enzymatically inactive members [2].

Phosphorylation by Ser/Thr/Tyr protein kinases plays a crucial role in cellular signal transduction. A canonical kinase domain consists of 12 subdomains containing few conserved residues of functional importance. Subdomains I, II, VIB, and VII are considered to be the most important ones. Subdomain I includes *β*-turn structure with 2 or 3 glycine residues (G-X-G-X-X-G) while subdomain II comprises an invariant lysine participating in anchoring and orienting the ATP (Adenosine Tri Phosphate). Subdomain VIB contains catalytic loop with a key aspartate [D] residue that mediates the transfer of a phosphate group from ATP to the appropriate substrate. The D residue of DFG motif in subdomain VII ligates Mg2+ which in turn interacts with *β* and *γ* phosphates of ATP [3]. Roles of these residues in protein kinases are well established. The catalytic residues of the protein kinases are usually highly conserved to maintain their ability for efficient cellular signal transduction. However, there have been few reports of proteins with substitutions/deletion at essential catalytic sites. Among these functionally important residues in a Ser/Thr/Tyr kinase, the aspartate residue in subdomain VIB acting as catalytic base seems to be most important as we are not aware of a properly functional kinase which lacks this residue.

Although the importance of protein kinases has long been recognized, studies on protein kinase homologues lacking catalytic residue/residues are more recent. Several studies on repertoire of kinases in various organisms have revealed presence of enzymatically inactive homologues of protein kinases [4–6] which lack catalytic function and instead serve as scaffolds or kinase substrates. Boudeau and colleagues have discussed roles of human kinase-like proteins in regulating diverse cellular processes [7].

Despite considerable sequence similarity to enzymatically active protein kinases, Protein Kinase-Like Nonkinase (PKLNK—also referred as Kinase Homology Domain—KHD in some of the earlier publications) domains lacking key residues thought to have regulatory roles. Some examples of proteins containing such domains which lack catalytic base aspartate are a PKLNK domain tethered to a tyrosine kinase domain in Janus Kinase (JAK) and membrane guanylyl cyclases (or particulate guanylyl cyclase) in which a regulatory PKLNK domain is situated N-terminal to the guanylyl cyclase domain [8–10]. PKLNK domain in JAK is thethered to functional kinase domain; however in guanylyl cyclases (GC), a functional kinase domain is absent, and the PKLNK is tethered to a cyclase domain. PKLNK domain of Guanylyl cyclase-A serves as an important mediator in transducing the ligand-induced signals to activate the catalytic cyclase domain of the receptor. Deletion of PKLNK domain from GC-A, -B, and -C resulted in constitutive activation of these enzymes [11, 12] and is shown to act as a repressor of the catalytic domain in the basal state [13]. The PKLNK of guanylyl cyclase-A (Natriuretic peptide receptor A) is more closely related to protein tyrosine kinase than protein serine/threonine kinase [11, 12, 14]. PKLNK in receptor guanylyl cyclase provides a critical structural link between the extracellular domain and the catalytic domain in regulating the activity of this family of receptor. Modeling of the PKLNK of human GC-C indicates that it can adopt a structure similar to that of tyrosine kinases [15]. There are many other protein kinase-like domains which lack other catalytically important residues, though playing important role as regulatory proteins, for example, “dead” RTK-ErbB3 [16], OTK (Off Track Kinase), WNK (with no lysine kinase), Tribbles, giant muscle protein titin (present in vertebrates), HER3, CCK-4 (Colon Carcinoma Kinase-4), Eph (Erythropoietin-producing hepatocyte) family of receptor tyrosine kinase, h-Ryk/d-Derailed, integrin-linked kinase (ILK) [17], and so forth. Recently, crystal structure of first PKLNK, VRK3 (a member of the vaccinia-related kinase family), which lacks aspartate in the catalytic loop has been reported [18] which revealed that it cannot bind ATP because of residue substitutions in the binding pocket, compared to ATP binding homologues. However, VRK3 still shares prominent structural similarity with enzymatically active protein kinase.

In the past, our group has reported presence of ABC1, RIO1, and kinases in archaea and bacteria that share significant similarity with Ser/Thr/Tyr kinase family [19]. The sequences of these protein kinases were examined for the presence of catalytic aspartate in the catalytic loop. Sixteen prokaryotes have been predicted to have at least one member lacking catalytic aspartate, and the total number of such sequences is 23. This study indicates that PKLNK has been evolved much before the divergence of prokaryote and eukaryote.

In the current analysis, we present a detailed analysis of the PKLNKs from four completely sequenced higher eukaryotes, namely, *Homo sapiens*, *Mus musculus*, *Rattus norvegicus*, and *Drosophila melanogaster*. An attempt has been made to classify these PKLNKs based upon their amino acid sequences and domain tethering preference in order to understand molecular basis of evolution and functions of these proteins.

## 2. Materials and Methods

In order to identify the repertoire of PKLNKs in various eukaryotic organisms PSI-BLAST [20] search was performed using traditional protein kinases as queries against the Nonredundant Data Base (NRDB) which is a database of protein amino acid sequences maintained at NCBI, USA. Hits were analyzed for the absence of catalytic base aspartate in the catalytic loop. In the PKLNKs, lacking catalytic aspartate, we further looked for the presence of other key-residues such as glycine in glycine-rich motif in the subdomain II, lysine and glutamic acid in the subdomain III, and DFG motif in subdomain VII. However we have considered all the kinase-like sequences lacking the catalytic base (Asp) residue for the present analysis.

The subfamily classification of these PKLNKs and recognition of other domains in the PKLNK domain containing multidomain proteins have been made using the procedures and protocols developed earlier in our group in connection with analysis of kinomes [4, 6, 21]. We have essentially employed multiple sensitive search and analysis methods like PSI-BLAST [20], RPS-BLAST [22], and HMMer [23] which match sequences to Hidden Markov Models (HMMs) of various families in Pfam (release 23) [24] to identify various domains in the multidomain sequences. Procedure such as PSI-BLAST has been used to detect homologues of noncatalytic kinase like domains using an E-value cut-off of 0.0001 that has been previously bench marked [25]. Hits lacking significant sequence similarity with the query have been further examined manually.

In the current analysis, we have identified a total 82 PKLNKs in the four organisms. CD-hits program [26, 27] was used in order to eliminate redundant sequences, which are indicated by 100% sequence identity. So the data set is devoid of redundant sequences.

CLUSTALW [28] has been used to align the nonenzymatic domains of 82 PKLNKs (see Table 1 in Supplementary Material available online at doi: 10.1155/2009/365637). Further, catalytic domain of the protein kinase and PKLNK domain from mouse have been aligned, and MEGA [29] was used to generate phylogenetic dendrograms.

Domain assignment to the other regions apart from the noncatalytic kinase domain of these PKLNKs has been carried out using HMMer search methods by querying each of the PKLNK against the 10340 protein families HMMs available in the Pfam database (http://pfam.sanger.ac.uk/). MulPSSM (Multiple PSSM) [30] approach was used further to assign domain to the region which has not been assigned using HMMer approach. Trans-membrane regions were detected using TMHMM [31].

## 3. Results and Discussion

In the current analysis, we have identified 82 PKLNKs. The main criteria used to detect these PKLNKs involve ensuring acceptable e-value with protein kinases and absence of catalytic base residue (Asp). There are 31 PKLNKs identified in *Homo sapiens* (Table 1), 18 PKLNKs in *Drosophila melanogaster* (Table 2), 13 PKLNKs in *Rattus norvegicus* (Table 3), and 20 PKLNKs in *Mus musculus* (Table 4). Although the catalytic Asp is absent in these sequences, we looked for the presence or absence of other key residues, characteristic of functional protein kinases, in the 82 identified PKLNKs. Glycine rich loop in the subdomain I (displaying consensus sequence G-X-G-X-X-G) contains at least two glycine residues in 26 gene products (see Supplementary Table 1). The phosphorylation of the activation segment is required for the activation of most protein kinases that contain an Arginine (R) preceding the catalytic base aspartate. We have essentially looked for the H-R-X motif (where X can be any residue but cannot be D) in all the 82 PKLNKs. There are 18 gene products which have “R” of H-R-X motif conserved (see Supplementary Table 1). We further checked for the presence of DFG and APE motifs in the activation loop and found that these motifs are not completely conserved in 82 PKLNKs identified so far. There are 12 and 49 protein gene products which have DFG and APE motif conserved, respectively, (see Supplementary Table 1).

**Figure 1:**
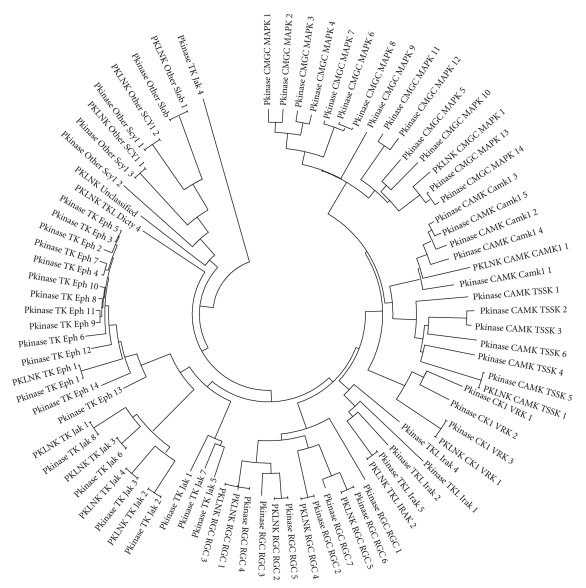
Phylogenetic tree representing catalytic kinase domain and PKLNK domains of mouse. Abbreviations followed in the figure are Pkinase, Protein kinase; PKLNK, Protein kinase-like nonkinase.

Though these PKLNKs lack the crucial aspartate in the catalytic loop, they are closely related to the functional protein kinases, in terms of the sequence similarity. Table 5 provides information on the closest protein kinase subfamily to which these PKLNKs belong to. Many of the PKLNKs are closely related to tyrosine kinase or tyrosine kinase-like group. Further, phylogenetic tree has been constructed considering PKLNK domain and catalytic domain of protein kinase subfamilies of mouse to which these PKLNKs from mouse are closely related (Figure 1). It has been observed that most of the PKLNKs from mouse are grouping to protein kinase subfamilies to which they closely belong to. This information provides a hint about the nearest evolutionary relation between PKLNKs and protein kinases. However, there are two PKLNKs from mouse, one of which is closely related to Tyrosine kinase-like group (gi|6005792), and the other one (gi|158635954) is not closely related to any of the known protein kinase subfamilies which are not grouping with their closest kinase subfamilies (Figure 1) suggesting that these two PKLNKs are evolutionary quite diverged.

### 3.1. Accessory Domains Tethered to the PKLNKs

**Figure 2:**
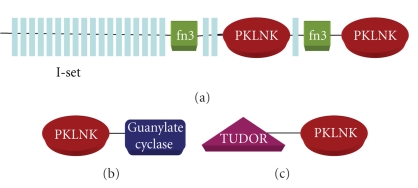
Unique domain architectures which are present in PKLNK and so far not reported in the protein kinase. Abbreviations followed in the figure are I-set, Immunoglobulin I-set; fn3, Fibronectin 3; PKLNK, Protein Kinase-Like Nonkinase.

In the multidomain proteins a given domain acts in conjunction with other domains which are tethered together in the same polypeptide chain and help in their regulation. Absence of catalytic aspartate in the active site reflects the noncatalytic activity of PKLNKs, but domain organization of a protein can give clues about putative functional roles. Domains tethered to PKLNKs suggest that though these PKLNKs lack key catalytic residues, they are involved in protein-protein interactions and might have important regulatory roles in signal transduction pathway. In the current analysis we have identified 19 different types of domains tethered to the PKLNK domain. Except 24 PKLNKs, majority of PKLNKs identified have accessory domains tethered to them (Table 6). The Pfam domains tethered to PKLNK domain and their frequency of occurrence are represented in Table 7. As can be seen in Table 7, the most commonly tethered domains are ANF receptor domain, Transmembrane domain and Guanylate cyclase domains. Interestingly most of the time it has been observed that all the three domains are present in the same polypeptide. There are some domain families which occur in repeats like Immunoglobulin I-set domain and HEAT domain which are mainly involved in cell-cell recognition, and protein-protein interactions, respectively, have also been found tethered to the PKLNK domain. Prediction of transmembrane domain has revealed occurrence of receptor PKLNKs which have most of the time single pass transmembrane region. Interestingly a drosophila protein (gi|21626698) has two PKLNK domains, many I-set (Immunoglobulin) repeats and fn3 domains which has been observed for the first time (Figure 2(a)) and not seen in any functional protein kinase. Our study has revealed that these two PKLNK domains are closely related to myosin light chain kinase subfamily of calcium/calmodulin dependent kinase group. There are a few PKLNKs which are closely related to receptor guanylate cyclase family of protein kinase which is characterized by extracellular ANF receptor domain. Interestingly some of these PKLNKs which are closely related to receptor guanylate cyclase subfamily of protein kinase do not have extracellular domain predicted in the N-terminal (Figure 2(b)) suggesting evolutionary paradigm.

Based upon the broad function, the domains tethered to PKLNK can be functionally categorized into four categories:

1. Domains which are mainly involved in ligand binding like ANF receptor, Receptor L domain, and Ephrin receptor ligand binding domain.

There are 17 gene products which have domains architecture similar to ANP receptor [12] in which ANF_receptor is followed by PKLNK which is followed by Guanylate cyclase domain. The ANF receptor is an extracellular ligand binding domain in a wide range of receptors [33]. Guanylate cyclase catalyses the formation of cyclic GMP (cGMP) from GTP which acts as intracellular messenger and regulates various cellular processes like smooth muscle relaxation, retinal phototransduction, regulation of ion channels, and so forth [34, 35]. The ephrin receptor ligand-binding domain (EPH_lbd) which binds to ephrin is a large family of receptor tyrosine kinases. Biochemical studies suggest that the multimerization of EPH_lbd modulates the cellular response and acts on actin cytoskeleton [36].

(2) Domains which are extracellular and involved in protein-protein interactions like I-set (Immunoglobulin like domain) and Fn3 (Fibronectin type III) domains.
(3) Domains involved in dimerization like Death domain, SAM (Sterile Alpha Motif) domain, and Furin-like domain.

Proteins containing death domains are well known to participate in the signaling events which regulate apoptosis [37] indicating role of PKLNK in apoptosis. Proteins containing SAM domains are involved in homo- and hetero oligomerization with other SAM domains and are involved in various developmental processes [38]. Furin-like domain is found tethered to receptor tyrosine kinase. It is rich in cysteine and involved in receptor aggregation.

(4) Domains involved in protein-protein interactions like Ank (Ankyrin repeats) and Heat repeats.

Ank is one of the most common protein-protein interaction modules which occur in large number of functionally diverse proteins. PKLNKs containing Ank repeat are likely to play role in diverse functions like signal transduction, ion transportation, transcription initiation, and so forth. Heat domain is 30–40 amino acid tandemly repeated domain. PKLNK containing Heat domain might have role in intracellular transport processes.

Apart from the domains discussed above there are some more accessory domains found tethered to the PKLNK domain which provide functional diversity to the PKLNKs. A human PKLNK (gi|18676872) has TBC domain and Rhodanese domain in the C-terminal. TBC domain is involved in GTPase signaling, and Rhodanese domain which shares evolutionary relationship with large family of protein is involved in cyanide detoxification [39]. Another human PKLNK (gi|17368698) has TUDOR domain N-terminal to the PKLNK domain which indicates its role in RNA binding [40] which has so far not seen tethered with protein kinase (Figure 2(c)).

A drosophila PKLNK (gi|17368346) has WSC domains N-terminal to the PKLNK domain which is likely to be an extracellular carbohydrate binding domains. At least three PKLNKs (gi|20869393, gi|21627748, gi|7020363) have PX domain N-terminal to the PKLNK domain which might have role in lipid signaling.

**Figure 3:**
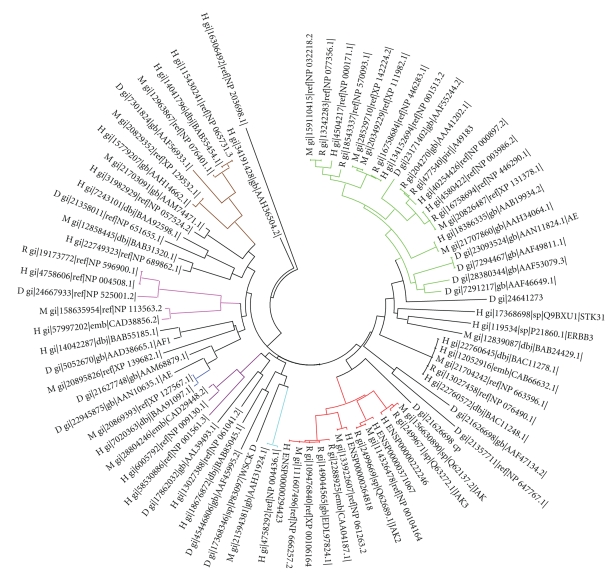
The dendrogram generated by considering the nonenzymatic protein kinase-like nonkinase domains. Various sequences having similar domain organization are represented in different colours: Green: PKLNK having Guanylate Cyclase (GC) domain in the C-terminus; Pink: Ankyrin repeat tethered to the PKLNK; Cyan: Ephrin_lbd, fn3, SterilAlpha Motif (SAM) tethered to the PKLNK; Purple: Death domain tethered to the PKLNK; Blue: Phox (PX) domain tethered to the PKLNK; Brown: Heat domain tethered to the PKLNK; Red: Src Homology 2 (SH2) tethered to the PKLNK.

Phylogenetic tree has been generated by considering the nonenzymatic PKLNK domains of these 82 PKLNKs (Figure 3) in which interestingly we have observed some clusters having similar domain organization. Some of the frequently found tethered domains have been represented in various colours. It can be noticed that, in general, PKLNKs with similar domains tethered are clustered together in Figure 3. There are few PKLNKs which have only one domain, and other parts of the sequences have not been assigned to any other Pfam domains. There are 20 PKLNKs which have guanylate cyclase domains tethered in the C-terminus (represented in green in Figure 3). There are 13 PKLNK sequences which have SH2 (Src homology 2) domain in the N-terminus (represented in red in Figure 3). SH2 domain functions as regulatory module of intracellular signaling cascade by interacting with the phosphopeptide. All of these SH2 containing PKLNKs except one (gi|2288925) have protein kinase domain tethered in the C-terminus. These 12 protein kinases domains are close homologues of protein tyrosine kinase 7 subfamily. This kind of domain architecture having SH2 domain followed by PKLNK which is followed by protein kinase domain has not been reported anywhere to the best of our knowledge. However, JAK1 (Janus kinase 1) has very similar domain combination in which apart from these three domains, FERM domain which, is involved in binding to cytokine receptors [32, 41] is present in the N-terminus [42].

The biological function of these PKLNKs might be in the regulation of tyrosine protein kinase activity.

Further, we have compared the domain structure of PKLNKs and their closest protein kinase subfamilies. Interestingly, we have observed that there are a few domain combinations which are unique to either PKLNKs or protein kinases. There are a few Pfam domains such as TUDOR and HNOBA (Heme NO binding associated) which have not been seen tethered to protein kinase domains so far. HNOBA domain is known to function as heme-dependent sensor for gaseous ligands and transduce diverse downstream signals across diverse organisms [43]. The domain structures which commonly occur between PKLNK and protein kinase have also been studied (Table 8).

### 3.2. Protein-Protein Interaction of Human PKLNKs

Understanding the biological roles of proteins in the cellular environment is the main aim of genome analysis. For almost all cellular processes in a living cell protein-protein interactions are of central importance. In the current section, we have focused on human PKLNKs. We have looked for the protein-protein interactions of PKLNKs using HPRD database (http://www.hprd.org/) [44]. At least 9 human PKLNKs are shown to interact with various other proteins (Table 9) and most of these proteins are signaling proteins and adapter proteins which module the cell signaling and play critical role in cell polarization, differentiation, cell adhesion, neuronal cell development, apoptosis, homeostasis, and so forth. Four of these nine PKLNKs which are closely related to receptor guanylate cyclase (RGC) family of protein kinase are reported to interact mainly with natriuretic peptide and guanylate cyclase. The protein-protein interaction informations obtained from HPRD emphasize role of PKLNKs in signaling.

## 4. Conclusions

This work represents functional analysis of noncatalytic PKLNKs across a data set of 82 PKLNKs from four higher eukaryotes. Our analysis has indicated that existence of noncatalytic PKLNKs is quite common. The fact that noncatalytic PKLNKs are well conserved between *Homo sapiens*, *Mus musculus*, *Rattus norvegicus*, and *Drosophila melanogaster* strongly argues against pseudogenes, as otherwise these would have been lost during the evolutionary time. Our study on PKLNKs suggests that most noncatalytic PKLNKs are derived from active protein kinase ancestors and have lost one or more of the critical catalytic residues within the active site which provides new insight into nature's way of eliciting new functions of PKLNKs. Based upon the domain tethering preferences we have classified PKLNKs into four main classes in which members of the two classes are receptor PKLNKs which are mainly involved in ligand binding and protein-protein interaction extracellularly while other two classes of PKLNKs have members which are cytoplasmic, and they are mainly involved in dimerization and protein-protein interaction in the cytoplasm. The phylogenetic analysis reveals function-based clustering of these PKLNKs. Conservation of some of the modular organization across the four organisms suggests their central role in the eukaryotic signaling pathway. Since many of the PKLNKs have other domains tethered to them and are involved in protein-protein interactions, one can speculate that though the kinase-like domain is nonenzymatic, they might have role in regulation and scaffolding. Some of these catalytically inactive members of PKLNKs which are close homologues of the receptor tyrosine kinase are shown to be over-expressed in cancer cells. Additional studies are required to determine precise function and role of these PKLNKs in tumorgenesis and its usefulness in the diagnosis of tumors. Domain organization of these PKLNKs revealed that some of the PKLNKs have new and hence unique domain organization so far not seen in any other family of gene products. 3D structure and biochemical analysis can further determine and explore the functional role of these PKLNKs. The presence of putative PKLNK in higher eukaryotes indicates that we have more to learn about cellular signaling involving these noncatalytic domains. Evolutionary history of these PKLNKs would be of particular interest. It is hoped that this analysis will provide a better understanding about the frequent occurrence of PKLNKs in different organisms and hence their function.

## Supplementary Material

Multiple sequence alignment of the PKLNK domains of human, drosophila, mouse and rat. The site of replacement of catalytic residue is highlighted in red.

## Figures and Tables

**Table 1:** List of 31 human PKLNKs is represented in the table. Substitutions of various important residues in the motifs which are generally conserved in the functional protein kinase are shown for each human PKLNK. “-” indicates deletion.

Gene code	“HRD” motif in the catalytic loop	Activation loop start	Activation loop end
gi|119534|sp|P21860	HRN	DFG	ALE
gi|6005792|ref|NP_009130.1	CGS	DFA	PEE
gi|4758292|ref|NP_004436.1	HRS	RLG	APE
gi|4758606|ref|NP_004508.1	PRH	- - -	APE
gi|14042287|dbj|BAB55185.1	HGN	**- - **S	APE
gi|15779207|gb|AAH14662.1	HNN	- - G	APE
gi|7020363|dbj|BAA91097.1	YGH	DLE	- - -
gi|7243101|dbj|BAA92598.1	HGN	GFD	APE
gi|4504217|ref|NP_000171.1	HGR	DHG	APE
gi|14041796|dbj|BAB55454.1	HNN	- - G	A - -
gi|18676872|dbj|BAB85045.1	HRA	KFG	APE
gi|17368698|sp|Q9BXU1	HGS	DF D	APE
gi|18386335|gb|AAB19934.2	HGR	DFG	APE
gi|12052916|emb|CAB66632.1	HRN	DFH	- - -
gi|22760572|dbj|BAC11248.1	HRN	DFH	APE
gi|4580422|ref|NP_003986.2	HGS	DYG	APE
gi|13027388|ref|NP_061041.2	HRS	- - G	SPE
gi|22749323|ref|NP_689862.1	HGK	GFE	SP **-**
gi|22760645|dbj|BAC11278.1	HRN	DFH	APE
gi|16306492|ref|NP_203698.1	Deletion	- - -	SPE
gi|115430241	HNN	- - G	A - -
gi|134152694	HGR	DYG	APE
gi|31982929	- - -	GYG	SMD
gi|34191428	HRN	D - -	APE
gi|40254426	HGN	DYG	APE
gi|57997202	HR **-**	SPG	APE
gi|58530886	HSN	- - -	PED
ENSP00000222246	HGN	DPG	APE
ENSP00000264818	HGN	DPG	APE
ENSP00000294423	HGN	DPG	APE
ENSP00000371067	HGN	DPG	PPE

**Table 2:** List of 18 drosophila PKLNKs. Substitutions of various important residues in the motifs which are generally conserved in the functional protein kinase are shown for each drosophila PKLNK. “-” indicates deletion.

Gene code	“HRD” motif in the catalytic loop	Activation loop start	Activation loop end
gi|17368346|sp|P83097	HRQ	VFG	APE
gi|21626698|gb|AAF47134.2	HMG	DFG	SPE
gi|17862032|gb|AAL39493.1	HGS	NFS	APE
gi|7301824|gb|AAF56933.1	HNN	- - -	SPE
gi|7291217|gb|AAF46649.1	HGK	DFG	- - -
gi|7294467|gb|AAF49811.1	HGA	DFG	APE
gi|21357711|ref|NP_647767.1	- - -	- - -	- - -
gi|5052670|gb|AAD38665.1	HGN	- - -	APE
gi|21627748|gb|AAM68879.1	YGH	- - -	AIE
gi|22945875|gb|AAN10635.1	HGH	- - G	- - -
gi|23093524|gb|AAN11824.1	HGA	DFG	APE
gi|23171402|gb|AAF55244.2	HGN	DFG	APE
gi|21358011|ref|NP_651655.1	HRN	DFC	APE
gi|113194917|gb|AAF51744.3	- - -	DLG	APE
gi|24667933|ref|NP_525001.2	H - -	- - -	SPE
gi|28380344|gb|AAF53079.3	HGN	DFG	APE
gi|45446806|gb|AAF45995.2	- - -	- - -	APE
gi|24641273	HNY	DPG	RNL

**Table 3:** List of 13 rat PKLNKs. Substitutions of various important residues in the motifs which are generally conserved in the functional protein kinase are shown for each rat PKLNK. “-” indicates deletion.

Gene code	“HRD” motif in the catalytic loop	Activation loop start	Activation loop end
gi|19173772|ref|NP_596900.1	PRH	- - -	APE
gi|13027458|ref|NP_076490.1	HRN	DFH	APE
gi|16758684|ref|NP_446283.1	HGR	DYG	APE
gi|477540|pir||A49183	HGN	DYG	APE
gi|13242283|ref|NP_077356.1	HGR	DHG	APE
gi|204270|gb|AAA41202.1	HGN	DYG	APE
gi|18543337|ref|NP_570093.1	HGR	DHG	APE
gi|16758694|ref|NP_446290.1	HGS	DYG	APE
gi|109476840|ref|XP_001061647.1	HGN	DPG	APE
gi|149044565|gb|EDL97824.1	HGN	DPG	APE
gi|2288925|emb|CAA04187.1	HGN	DLG	APE
gi|2499669|sp|Q62689.1	HGN	DPG	PPE
gi|2499671|sp|Q63272.1	HGN	DPG	APE

**Table 4:** List of 20 mouse PKLNKs. Substitutions of various important residues in the motifs which are generally conserved in the functional protein kinase are shown for each mouse PKLNK. “-” indicates deletion.

Gene code	“HRD” motif in the catalytic loop	Activation loop start	Activation loop end
gi|12839087|dbj|BAB24429.1	HRL	DFG	CPE
gi|21594381|gb|AAH31924.1	HRA	RLG	APE
gi|21703091|gb|AAM74471.1	HGN	- - -	- - -
gi|21704242|ref|NP_663596.1	HRN	DFH	APE
gi|21707860|gb|AAH34064.1	HGR	DFG	APE
gi|12858445|dbj|BAB31320.1	HRN	GFE	SPE
gi|20829352|ref|XP_129532.1	HNN	- - G	SPE
gi|12963867|ref|NP_076401.1	HNN	- - G	PPE
gi|20349229|ref|XP_111982.1	HGR	DYG	APE
gi|20826487|ref|XP_131378.1	HGS	DYG	APE
gi|20895826|ref|XP_139682.1	- - -	DFG	APQ
gi|20869393|ref|XP_127567.1	YGH	D LE	- - -
gi|158635954|ref|NP_113563.2	HRS	- - -	APE
gi|159110415|ref|NP_032218.2	HGR	DHG	APE
gi|28529710|ref|XP_142224.2	HGR	DYG	APE
gi|28804246|emb|CAD29448.2	CGN	DFA	PEE
gi|111607496|ref|NP_666257.2	HGN	DPG	APE
gi|114326478|ref|NP_001041642.1	HGN	DPG	PPE
gi|133922607|ref|NP_061263.2	HGN	DPG	APE
gi|156630890|sp|Q62137.2	HGN	DPG	APE

**Table 5:** List of PKLNK analysed. Information on number of residues and the nearest protein kinase subfamily to which they belong to has also been provided. Abbreviations followed in the table are RGC, Receptor guanylate cyclase; CAMK1, Ca^2+^/Calmodulin dependent protein kinase 1; TSSK, Testis-specific serine/Threonine kinase; MLCK, Myosin light chain kinase; Eph, Ephrin receptor; EGFR, Epidermal growth factor receptor; JakA, Janus kinase A; MLK, Mixed lineage kinase; Slob, SLOw Border; NRBP, Nuclear receptor binding protein; TBCK, TBC domain-containing kinase; VRK, Vaccinia-related kinase; IRAK, IL1 receptor associated kinase; CDK, Cyclin dependent kinase; MAPK, Mitogen activated kinase; WNK, With no K (Lysine).

PKLNK gene accession code	Number of residues	Closest subfamily of protein kinase
gi|40254426|ref|NP_000897.2|	1061	RGC
gi|477540|pir||A49183	333	RGC
gi|4580422|ref|NP_003986.2|	1047	RGC
gi|20826487|ref|XP_131378.1|	477	RGC
gi|16758694|ref|NP_446290.1|	1047	RGC
gi|204270|gb|AAA41202.1|	1057	RGC
gi|23093524|gb|AAN11824.1|AE003537_3	1272	RGC
gi|7294467|gb|AAF49811.1|	1172	RGC
gi|7291217|gb|AAF46649.1|	1076	RGC
gi|23171402|gb|AAF55244.2|	1417	RGC
gi|4504217|ref|NP_000171.1|	1103	RGC
gi|159110415|ref|NP_032218.2|	1108	RGC
gi|13242283|ref|NP_077356.1|	1108	RGC
gi|134152694|ref|NP_001513.2|	1108	RGC
gi|28529710|ref|XP_142224.2|	1372	RGC
gi|20349229|ref|XP_111982.1|	625	RGC
gi|16758684|ref|NP_446283.1|	1108	RGC
gi|18543337|ref|NP_570093.1|	1110	RGC
gi|28380344|gb|AAF53079.3|	1163	RGC
gi|18386335|gb|AAB19934.2|	1073	RGC
gi|21707860|gb|AAH34064.1|	754	RGC
gi|22760572|dbj|BAC11248.1|	501	CAMK1
gi|22760645|dbj|BAC11278.1|	470	CAMK1
gi|12052916|emb|CAB66632.1|	473	CAMK1
gi|21704242|ref|NP_663596.1|	512	CAMK1
gi|13027458|ref|NP_076490.1|	504	CAMK1
gi|12839087|dbj|BAB24429.1|	292	TSSK
gi|21626698|gb|AAF47134.2|	3197	MLCK
gi|4758292|ref|NP_004436.1|	1006	Eph
gi|21594381|gb|AAH31924.1|	1014	Eph
gi|17368346|sp|P83097|WSCK_DROME	809	Eph
gi|119534|sp|P21860.1|ERBB3_HUMAN	1342	EGFR
gi|24641273	1177	JakA
ENSP00000294423	1156	JakA
gi|133922607|ref|NP_061263.2|	1184	JakA
gi|111607496|ref|NP_666257.2|	1153	JakA
gi|149044565|gb|EDL97824.1|	1153	JakA
gi|109476840|ref|XP_001061647.1|	1198	JakA
gi|2288925|emb|CAA04187.1|	866	JakA
ENSP00000371067	1132	JakA
gi|114326478|ref|NP_001041642.1|	1132	JakA
gi|2499669|sp|Q62689.1|JAK2_RAT	1132	JakA
gi|156630890|sp|Q62137.2|JAK3_MOUSE	1100	JakA
gi|2499671|sp|Q63272.1|JAK3_RAT	1100	JakA
ENSP00000222246	1131	JakA
ENSP00000264818	1187	JakA
gi|5052670|gb|AAD38665.1|AF145690_1	637	NRBP
gi|14042287|dbj|BAB55185.1|	535	NRBP
gi|21358011|ref|NP_651655.1|	835	SCY1
gi|7301824|gb|AAF56933.1|	873	SCY1
gi|14041796|dbj|BAB55454.1|	707	SCY1
gi|115430241|ref|NP_065731.3|	808	SCY1
gi|12963867|ref|NP_076401.1|	806	SCY1
gi|15779207|gb|AAH14662.1|	688	SCY1
gi|7243101|dbj|BAA92598.1|	796	SCY1
gi|20829352|ref|XP_129532.1|	735	SCY1
gi|113194917|gb|AAF51744.3|	2352	WNK
gi|21627748|gb|AAM68879.1|	646	Slob
gi|22945875|gb|AAN10635.1|AE003618_7	638	Slob
gi|7020363|dbj|BAA91097.1|	649	Slob
gi|20869393|ref|XP_127567.1|	582	Slob
gi|45446806|gb|AAF45995.2|	819	TBCK
gi|18676872|dbj|BAB85045.1|	893	TBCK
gi|31982929|ref|NP_057524.2|	474	VRK
gi|21703091|gb|AAM74471.1|	453	VRK
gi|24667933|ref|NP_525001.2|	448	MLK
gi|4758606|ref|NP_004508.1|	452	MLK
gi|19173772|ref|NP_596900.1|	452	MLK
gi|58530886|ref|NP_001561.3|	625	IRAK
gi|6005792|ref|NP_009130.1|	596	IRAK
gi|28804246|emb|CAD29448.2|	609	IRAK
gi|12858445|dbj|BAB31320.1|	472	Dicty4
gi|22749323|ref|NP_689862.1|	471	Dicty4
gi|16306492|ref|NP_203698.1|	240	CDK
gi|20895826|ref|XP_139682.1|	216	MAPK
gi|17862032|gb|AAL39493.1|	346	STE20
gi|13027388|ref|NP_061041.2|	418	STE20
gi|17368698|sp|Q9BXU1|STK31_HUMAN	1019	Unclassified protein kinase
gi|21357711|ref|NP_647767.1|	790	Unclassified protein kinase
gi|34191428|gb|AAH36504.2|	700	Unclassified protein kinase
gi|57997202|emb|CAD38856.2|	1491	Unclassified protein kinase
gi|158635954|ref|NP_113563.2|	1450	Unclassified protein kinase

**Table 6:** List of 82 PKLNKs identified from human, mouse, rat, and drosophila. Their gene accession code and domain architecture are also provided. Abbreviations followed in the table are SH2, Src homology 2; PKLNK, Protein kinase-like nonkinase, Pkinase, Protein kinase; Guanylate_cyc, Guanylate cyclase; TM, Transmembrane; Ank, Ankyrin; SAM, Sterile alpha motif; PX, Phox; I-set, Immunoglobulin; fn3, Fibronectin type III; Ephrin_lbd, Ephrin receptor ligand binding domain.

Gene accession code	Domain name, boundary, and E-value
ENSP00000222246	SH2, 377 457 0.0066 * PKLNK, 822 1071 2e-27 *
ENSP00000264818	PKLNK, 589 866 4.9e-10 * Pkinase, 897 1172 1.2e-44 *
ENSP00000294423	SH2, 441 526 0.0016 * PKLNK, 583 847 1e-12 * Pkinase, 877 1151 2.1e-40 *
ENSP00000371067	SH2, 401 481 0.00012 * PKLNK, 545 805 2.6e-10 * Pkinase, 849 1123 4.4e-42 *
gi|109476840|ref|XP_001061647.1|	SH2, 486 570 0.0016 * PKLNK, 627 889 1.5e-12 * Pkinase, 919 1193 3.5e-39 *
gi|111607496|ref|NP_666257.2|	SH2, 441 525 0.0038 * PKLNK, 582 844 1.8e-12 * Pkinase, 874 1148 4.5e-39 *
gi|113194917|gb|AAF51744.3|	PKLNK, 444 650 2.4e-21 *
gi|114326478|ref|NP_001041642.1|	SH2, 401 481 0.00014 * PKLNK, 545 805 1.5e-09 * Pkinase, 849 1123 5.2e-43 *
gi|115430241|ref|NP_065731.3|	PKLNK, 29 259 4e-88 * HEAT, 383 419 0.0067 * HEAT, 501 537 3.2e-05 *
gi|119534|sp|P21860.1|ERBB3_HUMAN	Recep_L_domain, 55 167 6.7e-45 * Furin-like, 180 332 3.4e-79 * Recep_L_domain, 353 474 3.4e-46 * TM, o644 666i * PKLNK, 709 965 1.9e-24 *
gi|12052916|emb|CAB66632.1|	PKLNK, 24 258 2.2e-38 *
gi|12839087|dbj|BAB24429.1|	PKLNK, 25 289 6e-55 *
gi|12858445|dbj|BAB31320.1|	PKLNK, 195 461 5e-11 *
gi|12963867|ref|NP_076401.1|	PKLNK, 29 301 0.00098 * HEAT, 383 419 0.014 * HEAT, 501 537 0.00015 *
gi|13027388|ref|NP_061041.2|	PKLNK, 58 369 2e-28 *
gi|13027458|ref|NP_076490.1|	PKLNK, 24 286 9.9e-70 *
gi|13242283|ref|NP_077356.1|	ANF_receptor, 75 411 5.3e-59 * TM, o468 490i * PKLNK, 520 808 1.8e-09 * Guanylate_cyc, 874 1061 2.2e-92 *
gi|133922607|ref|NP_061263.2|	PKLNK, 589 863 1.7e-10 * Pkinase, 894 1166 4.5e-43 *
gi|134152694|ref|NP_001513.2|	ANF_receptor, 71 412 3.1e-83 * TM, o468 490i * PKLNK, 532 809 3.1e-14 * Guanylate_cyc, 875 1062 3.4e-92 *
gi|14041796|dbj|BAB55454.1|	PKLNK 29 259 9e-89 * HEAT, 383 419 0.0067 * HEAT, 501 537 3.2e-05 *
gi|14042287|dbj|BAB55185.1|	PKLNK, 81 327 3.2e-08 *
gi|149044565|gb|EDL97824.1|	SH2, 441 525 0.0016 * PKLNK, 582 844 1.5e-12 * Pkinase, 874 1148 3.5e-39 *
gi|156630890|sp|Q62137.2|JAK3_MOUSE	PKLNK, 818 1091 2.1e-37 *
gi|15779207|gb|AAH14662.1|	PKLNK, 18 245 0.00017 *
gi|58635954|ref|NP_113563.2|	Ank, 55 87 0.00011 * Ank, 88 120 0.018 * PKLNK, 251 503 2e-06 *
gi|159110415|ref|NP_032218.2|	ANF_receptor, 75 411 1.1e-56 * TM, o345 364i * PKLNK, 520 811 2.3e-09 * Guanylate_cyc, 874 1061 7e-92 *
gi|16306492|ref|NP_203698.1|	PKLNK, 4 230 8.7e-56 *
gi|16758684|ref|NP_446283.1|	ANF_receptor, 71 412 2.8e-74 * TM, o468 490i * PKLNK, 532 809 8.6e-11 * Guanylate_cyc, 875 1062 2.2e-92 *
gi|16758694|ref|NP_446290.1|	ANF_receptor, 44 400 5.9e-75 * TM, o456 478i * PKLNK, 534 786 5.3e-17 * Guanylate_cyc, 852 1038 5.6e-107 *
gi|17368346|sp|P83097|WSCK_DROME	TM, i12 34o * WSC, 42 115 3.8e-25 * fn3, 129 233 2e-05 * TM, o422 444i * PKLNK, 511 768 2.6e-10 *
gi|17368698|sp|Q9BXU1|STK31_HUMAN	TUDOR, 28 147 2e-31 * PKLNK, 739 972 3.6e-05 *
gi|17862032|gb|AAL39493.1|	PKLNK, 10 298 3.7e-11 *
gi|18386335|gb|AAB19934.2|	ANF_receptor, 53 386 3.9e-32 * TM, o432 454i * PKLNK, 497 745 1.1e-09 * Guanylate_cyc, 815 1002 1.5e-104 *
gi|18543337|ref|NP_570093.1|	ANF_receptor, 88 422 7e-71 * TM, o480 502i * PKLNK, 550 818 2.4e-11 * Guanylate_cyc, 884 1071 1.4e-90 *
gi|18676872|dbj|BAB85045.1|	PKLNK, 26 273 3.4e-18 * TBC, 463 673 1.1e-10 * Rhodanese, 776 883 9.2e-09 *
gi|19173772|ref|NP_596900.1|	Ank, 33 65 9.4e-09 * Ank, 66 98 1.6e-09 * Ank, 99 131 3.2e-07 * PKLNK, 193 449 1.5e-10 *
gi|20349229|ref|XP_111982.1|	PKLNK, 1 214 2e-08 * Guanylate_cyc, 306 521 1.2e-79 *
gi|204270|gb|AAA41202.1|	ANF_receptor, 50 412 1.3e-80 * PKLNK, 534 797 8.4e-13 * Guanylate_cyc, 863 1049 6e-109 *
gi|20826487|ref|XP_131378.1|	PKLNK, 1 216 7e-09 * Guanylate_cyc, 282 468 5.6e-107 *
gi|20829352|ref|XP_129532.1|	PKLNK, 18 245 8.9e-05 *
gi|20869393|ref|XP_127567.1|	PX, 17 122 2.4e-19 * PKLNK, 146 443 0.0012 *
gi|20895826|ref|XP_139682.1|	PKLNK, 4 206 3.7e-06 *
gi|21357711|ref|NP_647767.1|	PKLNK, 406 658 0.0018 *
gi|21358011|ref|NP_651655.1|	PKLNK, 31 314 5.7e-09 *
gi|21594381|gb|AAH31924.1|	TM, i13 32o * Ephrin_lbd, 34 227 4.5e-113 * fn3, 365 463 1.8e-06 * fn3, 481 565 1.3e-17 * TM, o590 612i * PKLNK, 663 908 7.4e-22 * SAM, 938 1005 1.6e-22 *
gi|21626698|gb|AAF47134.2|	I-set, 2 87 2.1e-06 * I-set, 102 195 6.9e-17 * I-set, 203 278 0.00011 * I-set, 292 382 7.6e-23 * I-set, 385 479 2.6e-05 * I-set, 483 574 2.6e-15 * I-set, 578 669 6.2e-11 * I-set, 673 765 7.1e-20 * I-set, 800 884 3.1e-10 * I-set, 893 993 0.00015 * I-set, 997 1087 2.5e-10 * I-set, 1092 1194 1.3e-05 * I-set, 1199 1290 2.1e-21 * I-set, 1297 1387 7.4e-24 * I-set, 1394 1485 7.3e-23 * I-set, 1498 1587 4.6e-21 * I-set, 1594 1683 1.1e-18 * I-set, 1696 1786 1.1e-21 * fn3, 1812 1898 9.9e-07 * I-set, 1951 2042 0.00043 * I-set, 2046 2136 7.1e-12 * PKLNK, 2165 2419 2.5e-48 * I-set, 2633 2723 1.2e-17 * fn3, 2727 2809 1.2e-12 * PKLNK, 2876 3130 1.7e-34 *
gi|21627748|gb|AAM68879.1|	PX, 17 122 7.9e-15 * PKLNK, 146 435 0.00023 *
gi|21703091|gb|AAM74471.1|	PKLNK, 145 439 e-128 *
gi|21704242|ref|NP_663596.1|	PKLNK, 24 286 3.4e-70 *
gi|21707860|gb|AAH34064.1|	TM, o114 136i * PKLNK, 176 426 3.7e-10 *Guanylate_cyc, 496 683 2e-99 *
gi|22749323|ref|NP_689862.1|	PKLNK, 209 466 1e-13 *
gi|22760572|dbj|BAC11248.1|	PKLNK, 24 286 7.8e-67 *
gi|22760645|dbj|BAC11278.1|	PKLNK, 24 286 3.4e-70 *
gi|2288925|emb|CAA04187.1|	SH2, 313 397 0.00056 * PKLNK, 454 716 3.2e-14 *
gi|22945875|gb|AAN10635.1|AE003618_7	PKLNK, 304 520 *
gi|23093524|gb|AAN11824.1|AE003537_3	ANF_receptor, 64 447 8e-33 * PKLNK, 610 869 4.4e-16 * Guanylate_cyc, 935 1121 1.5e-89 *
gi|23171402|gb|AAF55244.2|	ANF_receptor, 107 473 1.2e-83 * PKLNK, 608 877 1.8e-15 * Guanylate_cyc, 944 1130 9.7e-103 *
gi|24667933|ref|NP_525001.2|	Ank, 33 65 1.6e-07 * Ank, 66 98 1.4e-06 * Ank, 99 131 1.5e-08 * PKLNK, 192 446 6.9e-09 *
gi|2499669|sp|Q62689.1|JAK2_RAT	SH2, 401 481 8.6e-05 * PKLNK, 545 805 8.9e-10 * Pkinase, 849 1123 1.9e-43 *
gi|2499671|sp|Q63272.1|JAK3_RAT	PKLNK, 818 1091 2.7e-38 *
gi|28380344|gb|AAF53079.3|	ANF_receptor, 57 398 2e-37 * PKLNK, 529 798 3e-11 * Guanylate_cyc, 864 1050 3.3e-88 *
gi|28529710|ref|XP_142224.2|	ANF_receptor, 166 499 3.7e-62 * PKLNK, 647 926 1.5e-10 * Guanylate_cyc, 1018 1229 2.4e-72 *
gi|28804246|emb|CAD29448.2|	Death, 26 106 5.1e-18 * PKLNK, 178 456 1e-17 *
gi|31982929|ref|NP_057524.2|	PKLNK, 202 460 0.0082 *
gi|34191428|gb|AAH36504.2|	PKLNK, 48 317 7.9e-14 *
gi|40254426|ref|NP_000897.2|	ANF_receptor, 54 416 2.7e-79 * PKLNK, 538 801 3.7e-11 * Guanylate_cyc, 867 1053 7e-110 *
gi|4504217|ref|NP_000171.1|	ANF_receptor, 72 408 1.3e-55 * TM, o465 487i* PKLNK, 517 805 8.8e-10 * HNOBA, 718 870 0.009 * Guanylate_cyc, 871 1058 1.8e-89 *
gi|45446806|gb|AAF45995.2|	PKLNK, 18 263 1.2e-07 * TBC, 430 636 1.4e-08 *
gi|4580422|ref|NP_003986.2|	ANF_receptor, 44 400 2.7e-75 * PKLNK, 534 786 3.2e-18 * Guanylate_cyc, 852 1038 5.6e-107 *
gi|4758292|ref|NP_004436.1|	Ephrin_lbd, 18 217 4.5e-110 * fn3, 355 455 1.8e-06 * fn3, 473 557 3.2e-18 * TM, o582 604i * PKLNK, 655 900 1.1e-22 * SAM, 930 997 6e-22 *
gi|4758606|ref|NP_004508.1|	Ank, 33 65 9.4e-09 * Ank, 66 98 1.6e-09 * Ank, 99 131 3.2e-07 * PKLNK, 193 449 1.3e-10 *
gi|477540|pir||A49183	PKLNK, 16 250 7.9e-07 *
gi|5052670|gb|AAD38665.1|AF145690_1	PKLNK, 122 375 4.7e-09 *
gi|57997202|emb|CAD38856.2|	Ank, 55 87 0.0025 * Ank, 88 120 0.0056 * PKLNK, 251 503 1.6e-06 *
gi|58530886|ref|NP_001561.3|	Death, 14 94 2.9e-16 * PKLNK, 210 496 2.6e-11 *
gi|6005792|ref|NP_009130.1|	Death, 26 106 4.4e-18 * PKLNK, 165 443 3e-12 *
gi|7020363|dbj|BAA91097.1|	PX, 17 122 1.3e-16 * PKLNK, 146 449 0.0028 *
gi|7243101|dbj|BAA92598.1|	PKLNK, 4 190 2e-63 *
gi|7291217|gb|AAF46649.1|	ANF_receptor, 107 481 1.4e-73 * PKLNK, 555 794 3.9e-10 * Guanylate_cyc, 860 1046 1.2e-99 *
gi|7294467|gb|AAF49811.1|	ANF_receptor, 64 423 3.9e-35 * PKLNK, 573 832 4.4e-16 * Guanylate_cyc, 898 1084 1.5e-89 *
gi|7301824|gb|AAF56933.1|	PKLNK, 28 268 2e-82 * HEAT, 383 419 0.0027 *
gi|24641273	PKLNK, 892 1151 1.4e-35 *

**Table 7:** Frequency of domain families tethered to the PKLNKs.

Domain name	Number of gene products	Number of domains
ANF receptor	17	17
Guanylate cyclase	20	20
Protein kinase	9	9
Ankyrin	5	13
Fibronectin 3	4	7
Heat	4	7
Immunoglobulin I-set	1	21
Phox	3	3
TBC	2	2
Receptor L domain	1	2
Furin-like	1	1
Death	3	3
Ephrin receptor ligand binding domain	2	2
Sterile Alpha Motif	2	2
WSC	1	1
Rhodanese	1	1
Tudor	1	1
Transmembrane domain	13	15

**Table 8:** Common and unique domain structures of PKLNKs and protein kinases encoded in the data set of genomes. Abbreviations followed in the table are Mad3_BUB1_I, Mad3/BUB1 homology region 1; Pkinase, Protein kinase; PKLNK, Protein kinase-like nonkinase; TM, Transmembrane; SH2, Src-homology 2; I-set, Immunoglobulin; Ank, Ankyrin; fn3, Fibronectin type III; Ephrin_lbd, Ephrin receptor ligand binding domain; SAM, Sterile alpha motif; HNOBA, Heme NO binding associated; Guanylate_cyc, Guanylate cyclase.

Domain structure unique to PKLNKs	Domain structure unique to protein kinases	Domain structure common to protein kinases and PKLNKs
PKLNK, HEAT × 2	TM, ANF_receptor, Pkinase, Guanylate_cyc	SH2, PKLNK, Pkinase
Ank × 2, PKLNK	I-set × 2, Pkinase	Recep_L_domain, Furin-like, Recep_L_domain, TM, PKLNK
TUDOR, PKLNK	Mad3_BUB1_I, Pkinase	PKLNK, Pkinase
ANF_receptor, TM, PKLNK, HNOBA, Guanylate_cyc	Ank × 3, Pkinase	TM, WSC, fn3, TM, PKLNK
		ANF_receptor, TM, PKLNK, Guanylate_cyc
		PKLNK, TBC, Rhodanese
		PKLNK, Guanylate_cyc
		TM, Ephrin_lbd, fn3 × 2, TM, PKLNK, SAM
		I-set × 18, fn3, I-set × 2, PKLNK, I-set, fn3, PKLNK
		TM, PKLNK, Guanylate_cyc
		SH2, PKLNK
		ANF_receptor, PKLNK, Guanylate_cyc
		PKLNK, TBC
		Ephrin_lbd, fn3 × 2, TM, PKLNK, SAM
		Death, PKLNK
		PX, PKLNK
		PKLNK, HEAT

**Table 9:** List of PKLNKs which interact with a large number of proteins. The in vivo/in vitro protein-protein interaction data has been obtained from HPRD database [32].

PKLNK	Accession code of the interacting protein	Name	Role
NP_009130.1	NP_001560	Interleukin-1 receptor-associated kinase 1 isoform 1	Partially responsible for IL1-induced upregulation of the transcription factor NF-kappa B
	NP_001561	Interleukin-1 receptor-associated kinase 2	Participate in the IL1-induced upregulation of NF-kappa B
	NP_002459	Myeloid differentiation primary response gene 88	Functions as adapter protein in the association of IL-1 receptor associated kinase (IRAK) with the IL-1 receptor
	NP_067681	Toll-like receptor adaptor molecule 2	Involved in Toll receptor siganling
	NP_665802	TNF receptor-associated factor 6	Mediates signal transduction from members of the TNF receptor superfamily and from the members of the Toll/IL-1 family

NP_004436.1	NP_001035090	Myeloid/lymphoid or mixed-lineage leukemia	Regulates cell-cell adhesions downstream of Ras activation
	NP_002077	Growth factor receptor-bound protein 2 isoform 1	Binds epidermal growth factor receptor. Involved in signal transduction pathway
	NP_002961	Spermidine/spermine N1-acetyltransferase	Involved in the catabolic pathway of plymine metabolism
	NP_004084	Ephrin B2	Mediates developmental events, especially in the nervous system and in erythropoiesis
	NP_004432	Ephrin receptor EphB1 precursor	Mediates developmental events, especially in the nervous system and in erythropoiesis
	NP_005179	Cas-Br-M (murine) ecotropic retroviral transforming sequence	Adaptor protein for receptor protein-tyrosine kinases, positively regulates receptor protein-tyrosine kinase ubiquitination in a manner dependent upon its variant SH2 and RING finger domains
	NP_005198	v-crk sarcoma virus CT10 oncogene homolog	Plays a role in fibroblast transformation
	NP_058431	v-crk sarcoma virus CT10 oncogene homolog isoform a	Member of an adapter protein family that binds to several tyrosine-phosphorylated proteins
	NP_115500	Haloacid dehalogenase-like hydrolase domain containing 2	Hydrolase

NP_004508.1	AAM77350	LIMS2	Is a focal adhesion protein that associates with integrin-linked kinases and involved in protein-protein interactions at adhesion sites between cells and the extracellular matrix
	BAA18998	Paxillin gamma	A focal adhesion complex (FAC) which interacts with a wide array of molecules involved in managing the cells response to extracellular matrix components, growth factors, cell : cell interactions, and chemotatic signals.
	NP_000202	Integrin, beta 2 precursor	Cell-surface protein participates in cell adhesion as well as cell-surface mediated signaling
	NP_000203	Integrin beta chain, beta 3	Participates in cell adhesion as well as cell-surface mediated signaling
	NP_001003828	Parvin, beta isoform a	Actin-binding proteins associated with focal contacts
	NP_001744	Caveolin 1	Main component of the caveolae plasma membranes, links integrin subunits to the tyrosine kinase FYN which helps in cell cycle progression
	NP_002084	Glycogen synthase kinase 3 beta	Involved in energy metabolism, neuronal cell development, and body pattern formation
	NP_002471	Protein phosphatase 1, regulatory (inhibitor) subunit 12A	Regulates interaction of actin and myosin downstream of the guanosine triphosphatase Rho
	NP_002604	3-phosphoinositide dependent protein kinase-1 isoform 1	Phosphorylates and activates protein kinase B alpha and p70 S6 kinase
	NP_004978	LIM and senescent cell antigen-like domains 1	Adaptor protein which may play role in integrin-mediated cell adhesion pr spreading
	NP_005154	v-akt murine thymoma viral oncogene homolog 1	Mediator of growth factor-induced neuronal survival
	NP_060692	Parvin, alpha	Actin-binding protein associated with focal contacts
	NP_066932	Thymosin, beta 4	Plays a role in regulation of actin polymerization, cell proliferation, migration, and differentiation
	NP_071424	Parvin, gamma	Actin-binding proteins associated with focal contacts
	NP_110395	Integrin-linked kinase-associated protein phosphatase 2C	Regulates the kinase activity of integrin-linked kinase and participate in Wnt signaling
	NP_391987	Integrin beta 1 isoform 1C-1 precursor	Involved in cell adhesion and recognition in a variety of processes including embryogenesis, hemostasis, tissue repair, immune response, and metastatic diffusion of tumor cells.

NP_000171.1	NP_000400	Guanylate cyclase activator 1A (retina)	Activator of guanylate cyclase
	NP_002089	Guanylate cyclase activator 1B (retina)	Activator of guanylate cyclase
	NP_006263	S100 calcium-binding protein, beta	Involved in cell cycle progression and differentiation
	NP_009033	Guanylate cyclase activator 2B	Activator of guanylate cyclase receptor
	O75916	Regulator of G-protein signaling 9	Inhibits signal transduction by increasing the GTPase activity of G-protein alpha subunits, involved in phosphotransduction

NP_003986.2	NP_003986	Natriuretic peptide receptor B precursor	Primary receptor for C-type natriuretic peptide, which upon ligand binding exhibits greatly increased guanylyl cyclase activity
	NP_077720	Natriuretic peptide precursor C	Possesses potent natriuretic, diurectic, and vasodilating activities and are implicated in body fluid homeostasis and blood pressure control

NP_061041.2	NP_000446	Serine/threonine protein kinase 11	Regulates cell polarity and functions as a tumor suppressor
	NP_001158	Baculoviral IAP repeat-containing protein 4	Inhibits apoptosis through binding to tumor necrosis factor receptor-associated factors TRAF1 and TRAF2
	NP_057373	Calcium binding protein 39	Calcium binding
	NP_663304	Mitogen-activated protein kinase kinase kinase 7 isoform B	Mediates signal transduction induced by TGF beta and morphogenetic protein and controls variety of cell functions including transcription regulation and apoptosis
	NP_665802	TNF receptor-associated factor 6	Mediates signal transduction from members of the TNF receptor superfamily and from the members of the Toll/IL-1 family

NP_065731.3	NP_004636	Coilin	The protein encoded by this gene is an integral component of Cajal bodies (also called coiled bodies). Cajal bodies are nuclear suborganelles of varying number and composition that are involved in the posttranscriptional modification of small nuclear and small nucleolar RNAs.
	NP_036204	CD93 antigen precursor	Involved in intercellular adhesion and in the clearance of apoptotic cells
	NP_689494	SCY1-like 1 binding protein 1	Known to interact with SCY1-like family of kinase-like proteins

NP_001513.2	NP_001513.2	Guanylate cyclase 2F	Probably plays a specific functional role in the rods and/or cones of photoreceptors.
	NP_002089	Guanylate cyclase activator 1B (retina)	Activator of guanylate cyclase

NP_000897.2	NP_006249	Protein kinase, cGMP-dependent, type I isoform 2	Protein phosphorylation
	NP_002512	Natriuretic peptide precursor B preproprotein	Functions as cardiac hormone, has role in natriuresis, diuresis, vasorlaxation inhibition of rennin, and aldosterone secretion, and has a key role in cardiovascular homeostasis
	NP_000897	Natriuretic peptide receptor 1	Membrane bound guanylate cyclase that serves as the receptor for both atrial and brain natriuretic peptides
	NP_006163	Natriuretic peptide precursor A	Potent natriuretic, diuretic, and vasodilating activities and are implicated in body fluid homeostasis and blood pressure control
	NP_060649	Activating transcription factor 7 interacting protein	Modulates transcription regulation and chromatin formation
