# Supplementary material for: Classification of Nonenzymatic Homologues of Protein Kinases
Source: Comp Funct Genomics. 2009 Sep 28;2009:365637. doi: 10.1155/2009/365637 (PMC2754085; doi:10.1155/2009/365637)
Supplement: Supplementary file 1 — Multiple sequence alignment of the PKLNK domains of human, drosophila, mouse and rat. The site of replacement of catalytic residue is highlighted in red. [file 365637.f1.rtf]

Supplementary table 1: Multiple sequence alignment of the PKLNK domains of human, drosophila, mouse and rat. The site of replacement of catalytic residue is highlighted in red.


D_gi|5052670|gb|AAD38665.1|AF1      --------------------CVHLAMDTEEGVEVVW--------------
H_gi|14042287|dbj|BAB55185.1|       --------------------SAYLAMDTEEGVEVMW--------------
H_gi|22760645|dbj|BAC11278.1|       -------------------YDLGQVIKTEEFCEIFR--------------
H_gi|12052916|emb|CAB66632.1|       -------------------YDLGQVIKTEEFCEIFR--------------
M_gi|21704242|ref|NP_663596.1|      -------------------YDLGQVIKTEEFCEIFR--------------
R_gi|13027458|ref|NP_076490.1|      -------------------YDLGQVVKTEEFCEIFR--------------
H_gi|22760572|dbj|BAC11248.1|       -------------------YDLGQVIKTEEFCEIFR--------------
H_gi|4758292|ref|NP_004436.1|       -------------------IKIEEVIGTGSFGEVRQGRLQPRGR------
M_gi|21594381|gb|AAH31924.1|        -------------------IKIEEVIGAGSFGEVRRGRLQPRGR------
H_gi|119534|sp|P21860.1|ERBB3_      -------------------LRKLKVLGSGVFGTVHKGVWIPEGES-----
R_gi|149044565|gb|EDL97824.1|       -------------------IIQGEHLGRGTRTHIYSGTLLDYK---DDEG
R_gi|109476840|ref|XP_00106164      -------------------IIQGEHLGRGTRTHIYSGTLLDYK---DDEG
M_gi|111607496|ref|NP_666257.2      -------------------IIQGEHLGRGTRTHIYSGTLLDYK---DEEG
R_gi|2288925|emb|CAA04187.1|        -------------------IIQGEHLGRGTRTHIYSGTLLDYK---DDEG
H_ENSP00000294423                   -------------------LVQGEHLGRGTRTHIYSGTLMDYK---DDEG
H_ENSP00000264818                   -------------------ITQLSHLGQGTRTNVYEGRLRVEGSGDPEEG
M_gi|133922607|ref|NP_061263.2      -------------------ITQLSHLGQGTRTNVYEGLLRVGG---PDEG
M_gi|156630890|sp|Q62137.2|JAK      -------------------LEWHENLGHGSFTKIFRGRRREVVD-----G
R_gi|2499671|sp|Q63272.1|JAK3_      -------------------LEWHENLGHGSFTKIFHGHRREVVD-----G
H_ENSP00000222246                   -------------------LEWHENLGHGSFTKIYRGCRHEVVD-----G
M_gi|114326478|ref|NP_00104164      -------------------LIFNESLGQGTFTKIFKGVRREVGDY----G
R_gi|2499669|sp|Q62689.1|JAK2_      -------------------LIFNESLGQGTFTKIFKGVRREVGDY----G
H_ENSP00000371067                   -------------------LIFNESLGQGTFTKIFKGVRREVGDY----G
D_gi|23093524|gb|AAN11824.1|AE      -----------------------------TTIFTPIGLY-----------
D_gi|7294467|gb|AAF49811.1|         -----------------------------TTIFTPIGLY-----------
D_gi|28380344|gb|AAF53079.3|        ----------------ICRQSILVVGEPNKRSFTNIALF-----------
H_gi|40254426|ref|NP_000897.2|      -----------------SNYGSLLTTEGQFQVFAKTAYY-----------
R_gi|204270|gb|AAA41202.1|          -----------------SNYGSLLTTEGQFQVFAKTAYY-----------
R_gi|477540|pir||A49183             ----------------------QAVTETLAQGGTVTD-------------
H_gi|4580422|ref|NP_003986.2|       -----------------------------YQIFANTGHF-----------
R_gi|16758694|ref|NP_446290.1|      -----------------------------YQIFANTGHF-----------
M_gi|20826487|ref|XP_131378.1|      --------------------------------------------------
D_gi|23171402|gb|AAF55244.2|        -----------------------------RQIFIPVGMF-----------
D_gi|7291217|gb|AAF46649.1|         ------------------------FTSLPPQVYTTIGQF-----------
M_gi|159110415|ref|NP_032218.2      GGSSRKVVQGSRSSLATRSASDIRSVPSQPQESTNVGLY-----------
R_gi|13242283|ref|NP_077356.1|      GGSSRKVAQGSRSSLATRSTSDIRSVPSQPQESTNIGLY-----------
H_gi|4504217|ref|NP_000171.1|       GGTSRKVAQGSRSSLGARSMSDIRSGPSQHLDSPNIGVY-----------
R_gi|18543337|ref|NP_570093.1|      -GSPQSVIQGS-----------TRSVP-AFLEHTNVALY-----------
M_gi|28529710|ref|XP_142224.2|      ----FQIISEVQSGRSPRLSFSSGSLTPATYENSNIAIY-----------
M_gi|20349229|ref|XP_111982.1|      --------------------------------------------------
R_gi|16758684|ref|NP_446283.1|      ----FQIISEVQSGRSPRLSFSSGSLTPATYENSNIAIY-----------
H_gi|134152694|ref|NP_001513.2      ----FQITSEVQSGRSPRLSFSSGSLTPATYENSNIAIY-----------
H_gi|18386335|gb|AAB19934.2|        ---------------------------RDTIQRLRQCKY-----------
M_gi|21707860|gb|AAH34064.1|        ------------------------DRRRDTIQRVRQCKY-----------
H_gi|4758606|ref|NP_004508.1|       -------------------LNFLTKLNENHSGELWKGRW-----------
R_gi|19173772|ref|NP_596900.1|      -------------------LNFLAKLNENHSGELWKGRW-----------
D_gi|24667933|ref|NP_525001.2|      -------------------LDLHTKLSVTPSGETWRGRW-----------
H_gi|22749323|ref|NP_689862.1|      -------------------------LRENEVSTLYKGEYH----------
M_gi|12858445|dbj|BAB31320.1|       ----------------------WTKLKTSKMSTIYRGEYH----------
H_gi|6005792|ref|NP_009130.1|       -------------------FHKDFLIGEGEIFEVYRVEIQ----------
M_gi|28804246|emb|CAD29448.2|       -------------------FHKDFLIGEGEIFEVYRVDIR----------
H_gi|57997202|emb|CAD38856.2|       ------------------------------YMVMTNLVWN----------
M_gi|158635954|ref|NP_113563.2      ------------------------------YMVMTNLVWN----------
D_gi|21358011|ref|NP_651655.1|      --------YEVLEQVCTAGVGLMWKVYNGHKKSTKQEVS-----------
H_gi|7243101|dbj|BAA92598.1|        --------------------------------------------------
H_gi|14041796|dbj|BAB55454.1|       -----------------------WALHRGRKKATGSPVS-----------
H_gi|115430241|ref|NP_065731.3      -----------------------WALHRGRKKATGSPVS-----------
M_gi|12963867|ref|NP_076401.1|      -----------------------WILHRGRKKATGSAVS-----------
D_gi|7301824|gb|AAF56933.1|         ---------------------SIWTLHKAKRKTTLEEVS-----------
H_gi|15779207|gb|AAH14662.1|        -----------------------FTLPSGLAVYPAVLQD-----------
M_gi|20829352|ref|XP_129532.1|      -----------------------FTLPSGLAVYPAILQD-----------
D_gi|45446806|gb|AAF45995.2|        -----------------------FAKLHPGDVCGSNGLP-----------
H_gi|18676872|dbj|BAB85045.1|       ----------------------------------SNGLP-----------
H_gi|31982929|ref|NP_057524.2|      --------------------------------------------------
M_gi|21703091|gb|AAM74471.1|        ------WTLGALQIRDDQGILYEAEPTSAIPSESRTQKWR----------
H_gi|7020363|dbj|BAA91097.1|        WEVVEPLKDIGWRIRKKYFLMKIKNQPK---ERLVLS-------------
M_gi|20869393|ref|XP_127567.1|      WEVVEPLKDIGWRIRKKYFLMKIKNQPK---ERLVLS-------------
D_gi|21627748|gb|AAM68879.1|        WSLGGSMGAIGWRLRKHYFKVTTKPPEKSSNKQLVKSGSQTHQSKHFAAG
D_gi|22945875|gb|AAN10635.1|AE      ------LDEIGSRHGKNWFLVTDASVRTDRLQTLLPLPP-----------
D_gi|113194917|gb|AAF51744.3|       -------------------FKYDKEVGRGSFKTVYRGLD-----------
H_gi|58530886|ref|NP_001561.3|      -------------------FNQNRKISQGTFADVYRGHR-----------
D_gi|21626698|gb|AAF47134.2|        -------------------YDIGDELGRGTQGITYHAVERS---------
D_gi|17368346|sp|P83097|WSCK_D      -------------------LNVNDVIGDGRFGEIITGKVS----------
D_gi|21626698_cp                    -------------------YSFISEIARGEFSTIVKGIQKS---------
M_gi|12839087|dbj|BAB24429.1|       -------------------YEVGKIIGHGSYGTVYEAYYT----------
H_gi|13027388|ref|NP_061041.2|      -----------------YELQVEIGRGFDNLTSVHLARHTP---------
D_gi|17862032|gb|AAL39493.1|        -------------------YKLLEILKNGMIGTVYKAEDIN---------
H_gi|16306492|ref|NP_203698.1|      -------------------YTKIEKIGEGTYGVVYKGRHKT---------
D_gi|24641273                       -------------------YPDSISLSDDGMMFTMRGDWIQQSP------
H_gi|34191428|gb|AAH36504.2|        -------------------YQVLYQLNPGALGVNLVVEEMET--------
H_gi|17368698|sp|Q9BXU1|STK31_      --------------------------LSSKRPLVRSEVN-----------
M_gi|20895826|ref|XP_139682.1|      --------------------------------------------------
D_gi|21357711|ref|NP_647767.1|      ------------------ATTYRATHNTTGYKYCLRRIHGFRLQS-----
                                                                                      

D_gi|5052670|gb|AAD38665.1|AF1      ---------------NEVQYA----SLQELKSQE-EKMRQVFDNLLQLDH
H_gi|14042287|dbj|BAB55185.1|       ---------------NEVQFS----ERKNYKLQE-EKVRAVFDNLIQLEH
H_gi|22760645|dbj|BAC11278.1|       ---------------AKDKTTGKLHTCKKFQKRDGRKVRKAAKNEIGILK
H_gi|12052916|emb|CAB66632.1|       ---------------AKDKTTGKLHTCKKFQKRDGRKVRKAAKNEIGILK
M_gi|21704242|ref|NP_663596.1|      ---------------AKDKTTGKLHTCKKFQKRDGRKVRKAAKNEIGILK
R_gi|13027458|ref|NP_076490.1|      ---------------AKDKTTGKLHTCKKFQKRDGRKVRKAAKNEIGILK
H_gi|22760572|dbj|BAC11248.1|       ---------------AKDKTTGKLHTCKKFQKRDGRKVRKAAKNEIGILK
H_gi|4758292|ref|NP_004436.1|       ---------------REQTVAIQALWAG----GAESLQMT-FLGRAAVLG
M_gi|21594381|gb|AAH31924.1|        ---------------REQAVAIQALWAG----GAESLKMT-FLGRAALLG
H_gi|119534|sp|P21860.1|ERBB3_      ---------------IKIPVCIKVIEDK----SGRQSFQA-VTDHMLAIG
R_gi|149044565|gb|EDL97824.1|       IAEE-----------KKIKVILKVLDPS----HRDISLA--FFEAASMMR
R_gi|109476840|ref|XP_00106164      IAEE-----------KKIKVILKVLDPS----HRDISLA--FFEAASMMR
M_gi|111607496|ref|NP_666257.2      IAEE-----------KKIKVILKVLDPS----HRDISLA--FFEAASMMR
R_gi|2288925|emb|CAA04187.1|        IAEE-----------KKIKVILKVLDPS----HRDISLA--FFEAASMMR
H_ENSP00000294423                   TSEE-----------KKIKVILKVLDPS----HRDISLASAFFEAASMMR
H_ENSP00000264818                   KMDDEDPLVPGRDRGQELRVVLKVLDPS----HHDIALA--FYETASLMS
M_gi|133922607|ref|NP_061263.2      KVDNGCPPEPGGTSGQQLRVVLKVLDPS----HHDIALA--FYETASLMS
M_gi|156630890|sp|Q62137.2|JAK      ETHD-------------SEVLLKVMDSR----HRNCMES--FLEAASLMS
R_gi|2499671|sp|Q63272.1|JAK3_      ETHD-------------TEVLLKVMDSR----HQNCMES--FLEAASLMS
H_ENSP00000222246                   EARK-------------TEVLLKVMDAK----HKNCMES--FLEAASLMS
M_gi|114326478|ref|NP_00104164      QLHK-------------TEVLLKVLDKA----HRNYSES--FFEAASMMS
R_gi|2499669|sp|Q62689.1|JAK2_      QLHE-------------TEVLLKVLDKA----HRNYSES--FFEAASMMS
H_ENSP00000371067                   QLHE-------------TEVLLKVLDKA----HRNYSES--FFEAASMMS
D_gi|23093524|gb|AAN11824.1|AE      ---------------KGQLYAIKKVRKK----SVD--ITREMKKELKLLR
D_gi|7294467|gb|AAF49811.1|         ---------------KGQLYAIKKVRKK----SVD--ITREMKKELKLLR
D_gi|28380344|gb|AAF53079.3|        ---------------RGNIVAMKKIHKK----SVD--ITRSIRKELKLMR
H_gi|40254426|ref|NP_000897.2|      ---------------KGNLVAVKRVNRK----RIE--LTRKVLFELKHMR
R_gi|204270|gb|AAA41202.1|          ---------------KGNLVAVKRVNRK----RIE--LTRKVLFELKHMR
R_gi|477540|pir||A49183             ----------------GENITQRMWNRS----FQG--VTGYLKIDRNGDR
H_gi|4580422|ref|NP_003986.2|       ---------------KGNVVAIKHVNKK----RIE--LTRQVLFELKHMR
R_gi|16758694|ref|NP_446290.1|      ---------------KGNVVAIKHVNKK----RIE--LTRQVLFELKHMR
M_gi|20826487|ref|XP_131378.1|      ------------------------------------------------MR
D_gi|23171402|gb|AAF55244.2|        ---------------RKSKVAIKPVEVD----NVQGLLTRSLMLELKRMK
D_gi|7291217|gb|AAF46649.1|         ---------------KGERVAIKKVNVK----KVD--LTPQLLWEIKQAR
M_gi|159110415|ref|NP_032218.2      ---------------EGDWVWLKKFPGE-----HHMAIRPATKTAFSKLR
R_gi|13242283|ref|NP_077356.1|      ---------------EGDWVWLKKFPGE-----HHMAIRPATKMAFSKLR
H_gi|4504217|ref|NP_000171.1|       ---------------EGDRVWLKKFPGD-----QHIAIRPATKTAFSKLQ
R_gi|18543337|ref|NP_570093.1|      ---------------QGEWVWLKKFEAG-----TAPDLRPSSLSLLRKMR
M_gi|28529710|ref|XP_142224.2|      ---------------EGDWVWLKKFPPGD--FGDIKSIKSSASDVFEMMK
M_gi|20349229|ref|XP_111982.1|      ------------------------------------------------MK
R_gi|16758684|ref|NP_446283.1|      ---------------QGDWVWLKKFPPGD--FGDIKSIKSSASDVFEMMK
H_gi|134152694|ref|NP_001513.2      ---------------EGDWVWLKKFSLGD--FGDLKSIKSRASDVFEMMK
H_gi|18386335|gb|AAB19934.2|        ---------------VKKRVILKDLKHN------DGNFTEKQKIELNKLL
M_gi|21707860|gb|AAH34064.1|        ---------------DKKKVILKDLKHS------DGNFSEKQKIDLNKLL
H_gi|4758606|ref|NP_004508.1|       ---------------QGNDIVVKVLKVR----DWSTRKSRDFNEECPRLR
R_gi|19173772|ref|NP_596900.1|      ---------------QGNDIVVKVLKVR----DWSTRKSRDFNEECPRLR
D_gi|24667933|ref|NP_525001.2|      ---------------QKNDVVAKILAVR----QCTPRISRDFNEEFPKLR
H_gi|22749323|ref|NP_689862.1|      ----------------RAPVAIKVFKKLQAGSIAIVRQT--FNKEIKTMK
M_gi|12858445|dbj|BAB31320.1|       ----------------RSPVTIKVFNNPQAESVGIVRFT--FNDEIKTMK
H_gi|6005792|ref|NP_009130.1|       ----------------NLTYAVKLFKQEKKMQCKKHWKR--FLSELEVLL
M_gi|28804246|emb|CAD29448.2|       ----------------NQAYAVKLFKQEKKMQLKKHWKR--FLSELEVLL
H_gi|57997202|emb|CAD38856.2|       ----------------GSRVTVKELNLPTHPHCSRLRLADLLIAEQEHSS
M_gi|158635954|ref|NP_113563.2      ----------------RSRVTVKELNLPTRPHCSRLRLADLLIAEQEHSS
D_gi|21358011|ref|NP_651655.1|      ------------------VFVFEKKSLERWSKDDRETMLETLRRGVQQLT
H_gi|7243101|dbj|BAA92598.1|        --------------------------------------------------
H_gi|14041796|dbj|BAB55454.1|       ------------------IFVYDVKPGA-------EEQTQVAKAAFKRFK
H_gi|115430241|ref|NP_065731.3      ------------------IFVYDVKPGA-------EEQTQVAKAAFKRFK
M_gi|12963867|ref|NP_076401.1|      ------------------IFVYDVKPGA-------EEQTQVAKAAFKRLK
D_gi|7301824|gb|AAF56933.1|         ------------------VFVYDIRSGS-------DTKCELAKAALKRLK
H_gi|15779207|gb|AAH14662.1|        ------------------GKFASVFVYK-------RENEDKVNKAAKHLK
M_gi|20829352|ref|XP_129532.1|      ------------------GKCASVFVYK-------RENEDKVNKAAKHLK
D_gi|45446806|gb|AAF45995.2|        ------------------LTPNSIAILG----------------RAQKLK
H_gi|18676872|dbj|BAB85045.1|       ------------------LTPNSIKILG----------------RFQILK
H_gi|31982929|ref|NP_057524.2|      -----------------LKLDAKDGRLFNEQNFFQRAAKPLQVNKWKKLY
M_gi|21703091|gb|AAM74471.1|        ---------------FSLKLDSKDGRLFNEQNFFQRVAKPLQVNKWKKQF
H_gi|7020363|dbj|BAA91097.1|        -----------------------WADLG----PDKYLSDKDFQCLIKLLP
M_gi|20869393|ref|XP_127567.1|      -----------------------WADLG----PDKYLSDKDFQCLIKLLP
D_gi|21627748|gb|AAM68879.1|        SSNGSGHSIDAGTLDPGSEVVAEWLEYG----PDKFIDEKEIGGIMKSLM
D_gi|22945875|gb|AAN10635.1|AE      -------------------DCVAFEDLP----PNECAREILMELLGSLHH
D_gi|113194917|gb|AAF51744.3|       -----------------TLTGVPVAWCELLDKQVKKSERTRFREEADMLK
H_gi|58530886|ref|NP_001561.3|      ---------------HGKPFVFKKLRET--ACSSPGSIERFFQAELQICL
D_gi|21626698|gb|AAF47134.2|        ---------------SGDNYAAKIMYGR-------PELRPFMLNELEMMN
D_gi|17368346|sp|P83097|WSCK_D      ----------------TNDFARDCTLHVLCLDDLNGTTQAQLLRELRQLS
D_gi|21626698_cp                    ---------------TDTVVVAKILEVTD-------ENEDNVVAEFDNFK
M_gi|12839087|dbj|BAB24429.1|       --------------KQKVMVAVKIISKKK---ASEDYLNKFLPREIQVMK
H_gi|13027388|ref|NP_061041.2|      ---------------TGTLVTIKITNLEN-----CNEERLKALQKAVILS
D_gi|17862032|gb|AAL39493.1|        ----------------NKCLAVKKVSMD-----QPMEKLTLLFNEVLTVR
H_gi|16306492|ref|NP_203698.1|      ---------------TGQVVAMKKIRLE----SEEEGVPSTAIREISLLK
D_gi|24641273                       --------------VKDVSVTMKMLKSD--------GNFMEFFRLAQTWS
H_gi|34191428|gb|AAH36504.2|        ----------------KVKHVIKQVECMD------DHYASQALEELMPLL
H_gi|17368698|sp|Q9BXU1|STK31_      ----------------GQIILLKGYSVD---VDTEAKVIERAATYHRAWR
M_gi|20895826|ref|XP_139682.1|      --------------------------------------------EIQILL
D_gi|21357711|ref|NP_647767.1|      -------------TKCMTLVEMWKKLQHTNVVQLREVFTTKAFGDNSLVL
                                                                                      

D_gi|5052670|gb|AAD38665.1|AF1      QNI-VKFHRYWTDTQQAERPR---------VVFITEYMSSGSLKQFLKRT
H_gi|14042287|dbj|BAB55185.1|       LNI-VKFHKYWADIKEN-KAR---------VIFITEYMSSGSLKQFLKKT
H_gi|22760645|dbj|BAC11278.1|       MVK-HPNILQLVDVFVTRKEY---------FIFLELATGREVFDWILDQG
H_gi|12052916|emb|CAB66632.1|       MVK-HPNILQLVDVFVTRKEY---------FIFLELATGREVFDWILDQG
M_gi|21704242|ref|NP_663596.1|      MVK-HPNILQLVDVFVTRKEY---------FIFLELATGREVFDWILDQG
R_gi|13027458|ref|NP_076490.1|      MVK-HPNILQLVDVFVTRKEY---------FIFLELATGREVFDWILDQG
H_gi|22760572|dbj|BAC11248.1|       MVK-HPNILQLVDVFVTRKEY---------FIFLELATGREVFDWILDQG
H_gi|4758292|ref|NP_004436.1|       QFQ-HPNILRLEGVVTKSR----------PLMVLTEFMELGPLDSFLRQ-
M_gi|21594381|gb|AAH31924.1|        QFQ-HPNILRLEGVVTKSR----------PVMVLTELMELGPLDSFLRQ-
H_gi|119534|sp|P21860.1|ERBB3_      SLD-HAHIVRLLGLCPGS-----------SLQLVTQYLPLGSLLDHVRQ-
R_gi|149044565|gb|EDL97824.1|       QVS-HKHIVYLYGVCVRDV----------ENIMVEEFVEGGPLDLFMHR-
R_gi|109476840|ref|XP_00106164      QVS-HKHIVYLYGVCVRDV----------ENIMVEEFVEGGPLDLFMHR-
M_gi|111607496|ref|NP_666257.2      QVS-HKHIVYLYGVCVRDV----------ENIMVEEFVEGGPLDLFMHR-
R_gi|2288925|emb|CAA04187.1|        QVS-HKHIVYLYGVCVRDV----------ENIMVEEFVEGGPLDLFMHR-
H_ENSP00000294423                   QVS-HKHIVYLYGVCVRDV----------ENIMVEEFVEGGPLDLFMHR-
H_ENSP00000264818                   QVS-HTHLAFVHGVCVRGP----------ENIMVTEYVEHGPLDVWLRR-
M_gi|133922607|ref|NP_061263.2      QVS-HMHLAFLHGVCVRGS----------ENIIVTEFVEHGPLDVWLRR-
M_gi|156630890|sp|Q62137.2|JAK      QVS-YPHLVLLHGVCMAG-----------DSIMVQEFVYLGAIDMYLRK-
R_gi|2499671|sp|Q63272.1|JAK3_      QVS-YPHLVLLHGVCMAG-----------DSIMVQEFVYLGAIDTYLRK-
H_ENSP00000222246                   QVS-YRHLVLLHGVCMAG-----------DSTMVQEFVHLGAIDMYLRK-
M_gi|114326478|ref|NP_00104164      QLS-HKHLVLNYGVCVCGE----------ENILVQEFVKFGSLDTYLKK-
R_gi|2499669|sp|Q62689.1|JAK2_      QLS-HKHLVLNYGVCVCGE----------ENILVQEFVKFGSLDTYLKK-
H_ENSP00000371067                   KLS-HKHLVLNYGVCVCGD----------ENILVQEFVKFGSLDTYLKK-
D_gi|23093524|gb|AAN11824.1|AE      DAR-HDNICAFIGACTDPPN----------ICIISEYCTRGSLKDILEN-
D_gi|7294467|gb|AAF49811.1|         DAR-HDNICAFIGACTDPPN----------ICIISEYCTRGSLKDILEN-
D_gi|28380344|gb|AAF53079.3|        EVR-HENIINFIGASTDHGS----------VIIFTTYCARGSLEDVLAN-
H_gi|40254426|ref|NP_000897.2|      DVQ-NEHLTRFVGACTDPPN----------ICILTEYCPRGSLQDILEN-
R_gi|204270|gb|AAA41202.1|          DVQ-NEHLTRFVGACTDPPN----------ICILTEYCPRGSLQDILEN-
R_gi|477540|pir||A49183             DTD-FSLWD-------------------------------GSLQDILEN-
H_gi|4580422|ref|NP_003986.2|       DVQ-FNHLTRFIGACIDPPN----------ICIVTEYCPRGSLQDILEN-
R_gi|16758694|ref|NP_446290.1|      DVQ-FNHLTRFIGACIDPPN----------ICIVTEYCPRGSLQDILEN-
M_gi|20826487|ref|XP_131378.1|      DVQ-FNHLTRFIGACIDPPN----------ICIVTEYCPRGSLQDILEN-
D_gi|23171402|gb|AAF55244.2|        DLQ-HDHLVKFYGACLDQRR----------SFLLTEYCPKGSLQDILEN-
D_gi|7291217|gb|AAF46649.1|         DVS-HENTVRFVGACIDLPRP--------TVLILTEYCSRGSLKDVLEN-
M_gi|159110415|ref|NP_032218.2      ELR-HENVALYLGLFLAGTADSPATPGEGILAVVSEHCARGSLHDLLAQ-
R_gi|13242283|ref|NP_077356.1|      ELR-HENVALYLGLFLAGTADSPATPGEGILAVVSEHCARGSLHDLLAQ-
H_gi|4504217|ref|NP_000171.1|       ELR-HENVALYLGLFLARGAEGPAALWEGNLAVVSEHCTRGSLQDLLAQ-
R_gi|18543337|ref|NP_570093.1|      EMR-HENVTAFLGLFVG-----PEVS-----AMVLEHCARGSLEDLLRN-
M_gi|28529710|ref|XP_142224.2|      DLR-HENVNPLLGFFYDS----------GMFAIVSEFCSRRSLEDILTN-
M_gi|20349229|ref|XP_111982.1|      DLR-HENVNPLLGFFYDS----------GMFAIVSEFCSRRSLEDILTN-
R_gi|16758684|ref|NP_446283.1|      DLR-HENVNPLLGFFYDS----------GMFAIVSEFCSRRSLEDILTQ-
H_gi|134152694|ref|NP_001513.2      DLR-HENINPLLGFFYDS----------GMFAIVTEFCSRGSLEDILTN-
H_gi|18386335|gb|AAB19934.2|        QID-YYTLTKFYGTVKLDTM----------IFGVIEYCERGSLREVLNDT
M_gi|21707860|gb|AAH34064.1|        QSD-YYNLTKFYGTVKLDTR----------IFGVVEYCERGSLREVLNDT
H_gi|4758606|ref|NP_004508.1|       IFS-HPNVLPVLGACQSPPA--------PHPTLITHWMPYGSLYNVLHEG
R_gi|19173772|ref|NP_596900.1|      IFS-HPNVLPVLGACQAPPA--------PHPTLITHWMPYGSLYNVLHEG
D_gi|24667933|ref|NP_525001.2|      IFS-HPNILPIIGACNSP----------PNLVTISQFMPRSSLFSLLHGA
H_gi|22749323|ref|NP_689862.1|      KFE-SPNILRIFGICIDETV------TPPQFSIVMEYCELGTLRELLD--
M_gi|12858445|dbj|BAB31320.1|       KFD-SPNILRIFGICIDQTV------KPPEFSIVMEYCELGTLRELLD--
H_gi|6005792|ref|NP_009130.1|       LFH-HPNILELAAYFTET----------EKFCLIYPYMRNGTLFDRLQ--
M_gi|28804246|emb|CAD29448.2|       LFR-HPHILELAAYFTET----------EKLCLVYPYMSNGTLFDRLQ--
H_gi|57997202|emb|CAD38856.2|       KLR-HPYLLQLMAVCLSQ--------DLEKTRLVYERITIGTLFSVLHE-
M_gi|158635954|ref|NP_113563.2      NLR-HPNLLQLMAVCLSR--------DLEKIRLVYERITVGTLFSVLHE-
D_gi|21358011|ref|NP_651655.1|      KIR-HPHVLTVQHPLEESRDS---------LAFATEPVFASLANVVGDNV
H_gi|7243101|dbj|BAA92598.1|        ------------------------------------------SGDIGD--
H_gi|14041796|dbj|BAB55454.1|       TLR-HPNILAYID-GLETEKC---------LHVVTEAVTP--LGIYLKAR
H_gi|115430241|ref|NP_065731.3      TLR-HPNILAYID-GLETEKC---------LHVVTEAVTP--LGIYLKAR
M_gi|12963867|ref|NP_076401.1|      TLR-HPNILAYID-GLETEKC---------LHIVTEAVTP--LGTYLKAR
D_gi|7301824|gb|AAF56933.1|         TLR-HPSILQYLD-SLETDKM---------LYVATEAVDP--LGTYFSKL
H_gi|15779207|gb|AAH14662.1|        TLR-HPCLLRFLS-CTVEADG---------IHLVTERVQP--LEVALETL
M_gi|20829352|ref|XP_129532.1|      TLR-HPCLLRFLS-CTVEADG---------IHLVTERVQP--LEVALETL
D_gi|45446806|gb|AAF45995.2|        ELQ-DEHLCQYLDVIRGKHER---------TIVVSEYLGL----SLEDYA
H_gi|18676872|dbj|BAB85045.1|       TIT-HPRLCQYVDISRGKHER---------LVVVAEHCER----SLEDLL
H_gi|31982929|ref|NP_057524.2|      STP-LLAIPTCMGFGVHQDK----------YRFLVLPSLGRSLQSALDVS
M_gi|21703091|gb|AAM74471.1|        LLP-LLAIPTCIGFGIHQDK----------YRFLVFPSLGRSLQSALDDN
H_gi|7020363|dbj|BAA91097.1|        SCL-HPYIYRVTFATANESS----------ALLIRMFNEKGTLKDLIYKA
M_gi|20869393|ref|XP_127567.1|      SCV-HPYIYRVTFATASESS----------ALLIRAFNEKGTLKDLIYKA
D_gi|21627748|gb|AAM68879.1|        GLQ-HPHIEPVLLAAHTENG----------CLVIRKFHKHGTLKDVLCMA
D_gi|22945875|gb|AAN10635.1|AE      PYI-YPVLDLGFLRNSSYNY----------ACLVTPFNSRGSLKDLIYKA
D_gi|113194917|gb|AAF51744.3|       KLQ-HPNIVRFYTYWEFPIGR------KKNIVLVTELMLSGTLKSVKIG-
H_gi|58530886|ref|NP_001561.3|      RCC-HPNVLPVLGFCAARQFH----------SFIYPYMANGSLQDRLQGQ
D_gi|21626698|gb|AAF47134.2|        TFN-HKNLIRPYDAYDTDRS----------VTLIMELAAGGELVRDNLLR
D_gi|17368346|sp|P83097|WSCK_D      QLKRQEHLLDFYGVSASPDW----------FYLIFEQQRMSLKRKLVESR
D_gi|21626698_cp                    TLR-HERIPALFSAYKPLNVP--------IAIFVMEKLQGADVLTYFSSR
M_gi|12839087|dbj|BAB24429.1|       VLR-HKYLINFYQAIETTSR----------VYIILELAQGGDVLEWIQR-
H_gi|13027388|ref|NP_061041.2|      HFFRHPNITTYWTVFTVGS----------WLWVISPFMAYGSASQLLRTY
D_gi|17862032|gb|AAL39493.1|        RLQ-HRNINTIVSCFLYKQY----------VYLTYKFMCFGNCEVLLKN-
H_gi|16306492|ref|NP_203698.1|      ELR-HPNIVSLQDVLMQDSR----------LYLIFEFLSMDLKKYLDSIP
D_gi|24641273                       LIQ-SPQFLKLYGLTLADP-----------YTMVMEYSRYGPLNKFLHS-
H_gi|34191428|gb|AAH36504.2|        KLR-HAHISVYQELFITWNGEIS----SLYLCLVMEFNELSFQEVIEDKR
H_gi|17368698|sp|Q9BXU1|STK31_      EAEGDSGLLPLIFLFLCKSDP--------MAYLMVPYYPRANLNAVQAN-
M_gi|20895826|ref|XP_139682.1|      RFR-HENVIGIRDILRAPTLEAK------KDVYIVQNLMETDLYKLLKN-
D_gi|21357711|ref|NP_647767.1|      VYDYHPGSQTLLAKYFTPAPETNG----YTDPFQGEARPFSHKSNMQRTS
                                                                                      

D_gi|5052670|gb|AAD38665.1|AF1      -------------KRNAKRLPLESWRRWCTQILSALSYLHS--------C
H_gi|14042287|dbj|BAB55185.1|       -------------KKNHKTMNEKAWKRWCTQILSALSYLHS--------C
H_gi|22760645|dbj|BAC11278.1|       -------------YYSERDT-----SNVVRQVLEAVAYLHS--------L
H_gi|12052916|emb|CAB66632.1|       -------------YYSERDT-----SNVVRQVLEAVAYLHS--------L
M_gi|21704242|ref|NP_663596.1|      -------------YYSERDT-----SNVVRQVLEAVAYLHS--------L
R_gi|13027458|ref|NP_076490.1|      -------------YYSERDT-----SNVVRQVLEAVAYLHS--------L
H_gi|22760572|dbj|BAC11248.1|       -------------YYSERDT-----SNVVRQVLEAVAYLHS--------L
H_gi|4758292|ref|NP_004436.1|       ---------------REGQFSSLQLVAMQRGVAAAMQYLSS-------FA
M_gi|21594381|gb|AAH31924.1|        ---------------REGQFSSLQLVAMQRGVAAAMQYLSS-------FA
H_gi|119534|sp|P21860.1|ERBB3_      ---------------HRGALGPQLLLNWGVQIAKGMYYLEE-------HG
R_gi|149044565|gb|EDL97824.1|       ---------------KSDALTTPWKFKVAKQLASALSYLED-------KD
R_gi|109476840|ref|XP_00106164      ---------------KSDALTTPWKFKVAKQLASALSYLED-------KD
M_gi|111607496|ref|NP_666257.2      ---------------KSDALTTPWKFKVAKQLASALSYLED-------KD
R_gi|2288925|emb|CAA04187.1|        ---------------KSDALTTPWKFKVAKQLASALSYLED-------KD
H_ENSP00000294423                   ---------------KSDVLTTPWKFKVAKQLASALSYLED-------KD
H_ENSP00000264818                   ---------------ERGHVPMAWKMVVAQQLASALSYLEN-------KN
M_gi|133922607|ref|NP_061263.2      ---------------QRGQVPMTWKMVVAQQLASALSYLED-------KN
M_gi|156630890|sp|Q62137.2|JAK      ---------------RGHLVSASWKLQVTKQLAYALNYLED-------KG
R_gi|2499671|sp|Q63272.1|JAK3_      ---------------RGHLVPASWKLQVTKQLAYALNYLED-------KG
H_ENSP00000222246                   ---------------RGHLVPASWKLQVVKQLAYALNYLED-------KG
M_gi|114326478|ref|NP_00104164      ---------------NKNSINILWKLGVAKQLAWAMHFLEE-------KS
R_gi|2499669|sp|Q62689.1|JAK2_      ---------------NKNSINILWKLGVAKQLAWAMHFLEE-------KS
H_ENSP00000371067                   ---------------NKNCINILWKLEVAKQLAWAMHFLEE-------NT
D_gi|23093524|gb|AAN11824.1|AE      ---------------EDVKLDNMFIASMVADIIRGVIYLHD------SPI
D_gi|7294467|gb|AAF49811.1|         ---------------EDVKLDNMFIASMVADIIRGVIYLHD------SPI
D_gi|28380344|gb|AAF53079.3|        ---------------EDLHLDHMFISSLVSDILKGMIYLHD------SEI
H_gi|40254426|ref|NP_000897.2|      ---------------ESITLDWMFRYSLTNDIVKGMLFLHN------GAI
R_gi|204270|gb|AAA41202.1|          ---------------ESITLDWMFRYSLTNDIVKGMLFLHN------GAI
R_gi|477540|pir||A49183             ---------------ESITLDWMFRYSLTNDIVKGMLFLHN------GAI
H_gi|4580422|ref|NP_003986.2|       ---------------DSINLDWMFRYSLINDLVKGMAFLHN------SII
R_gi|16758694|ref|NP_446290.1|      ---------------DSINLDWMFRYSLINDLVKGMAFLHN------SII
M_gi|20826487|ref|XP_131378.1|      ---------------DSINLDWMFRYSLINDLVKGMAFLHN------SII
D_gi|23171402|gb|AAF55244.2|        ---------------EQFQLDWMFRLSLMHDIVRGMQFLHS------SDI
D_gi|7291217|gb|AAF46649.1|         ---------------EAIELDWNFRMSLIHDIVKGMNYLHN------SDV
M_gi|159110415|ref|NP_032218.2      ---------------REIKLDWMFKSSLLLDLIKGMRYLHH------RGV
R_gi|13242283|ref|NP_077356.1|      ---------------RDIKLDWMFKSSLLLDLIKGMRYLHH------RGV
H_gi|4504217|ref|NP_000171.1|       ---------------REIKLDWMFKSSLLLDLIKGIRYLHH------RGV
R_gi|18543337|ref|NP_570093.1|      ---------------EDLRLDWTFKASLLLDLIRGLRYLHH------RHF
M_gi|28529710|ref|XP_142224.2|      ---------------DDVKLDWMFKSSLLLDLIKGMKYLHH------REF
M_gi|20349229|ref|XP_111982.1|      ---------------DDVKLDWMFKSSLLLDLIKGMKYLHH------REF
R_gi|16758684|ref|NP_446283.1|      ---------------DDVKLDWMFKSSLLLDLIKGMKYLHH------REF
H_gi|134152694|ref|NP_001513.2      ---------------QDVKLDWMFKSSLLLDLIKGMKYLHH------REF
H_gi|18386335|gb|AAB19934.2|        I-----------SYPDGTFMDWEFKISVLYDIAKGMSYLHS------SKT
M_gi|21707860|gb|AAH34064.1|        I-----------SYPDGTFMDWEFKISVLNDIAKGMSYLHS------SKI
H_gi|4758606|ref|NP_004508.1|       ---------------TNFVVDQSQAVKFALDMARGMAFLHTL-----EPL
R_gi|19173772|ref|NP_596900.1|      ---------------TNFVVDQSQAVKFALDMARGMAFLHTL-----EPL
D_gi|24667933|ref|NP_525001.2|      ---------------TGVVVDTSQAVSFALDVARGMAFLHSL-----ERI
H_gi|22749323|ref|NP_689862.1|      ------------REKD---LTLGKRMVLVLGAARGLYRLHH-------SE
M_gi|12858445|dbj|BAB31320.1|       ------------REKD---LTMSVRSLLVLRAARGLYRLHH-------SE
H_gi|6005792|ref|NP_009130.1|       ------------CVGDTAPLPWHIRIGILIGISKAIHYLHN-------VQ
M_gi|28804246|emb|CAD29448.2|       ------------CTNGTTPLSWHVRISVLIGIAKAIQYLHN-------TQ
H_gi|57997202|emb|CAD38856.2|       ------------RRSQFPVLHMEVIVHLLLQISDALRYLHF-------QG
M_gi|158635954|ref|NP_113563.2      ------------RRSQFPVLHMEVIVHLLLQVADALIYLHS-------RG
D_gi|21358011|ref|NP_651655.1|      --------------RSEKKLYDVEIRHGLLQLFDGLQFLHQ---------
H_gi|7243101|dbj|BAA92598.1|        -----------------YKLYDVETKYGLLQVSEGLSFLHS---------
H_gi|14041796|dbj|BAB55454.1|       --------------VEAGGLKELEISWGLHQIVKALSFLVN---------
H_gi|115430241|ref|NP_065731.3      --------------VEAGGLKELEISWGLHQIVKALSFLVN---------
M_gi|12963867|ref|NP_076401.1|      --------------AEAGGLKEQELSWGLHQIVKALSFLVN---------
D_gi|7301824|gb|AAF56933.1|         --------------GSDNVQKGLYLAWGIFQITRALSFLNN---------
H_gi|15779207|gb|AAH14662.1|        --------------SS------AEVCAGIYDILLALIFLHD---------
M_gi|20829352|ref|XP_129532.1|      --------------SP------AEVCAGIYDILLALIFLHD---------
D_gi|45446806|gb|AAF45995.2|        --------------MRHPPLAIAQILRIFYQVACGINVLS----------
H_gi|18676872|dbj|BAB85045.1|       --------------RERKPVSCSTVLCIAFEVLQGLQYMN----------
H_gi|31982929|ref|NP_057524.2|      ---------------PKHVLSERSVLQVACRLLDALEFLHEN--------
M_gi|21703091|gb|AAM74471.1|        ---------------PKHVVSERCVLQVACRLLDALEYLHEN--------
H_gi|7020363|dbj|BAA91097.1|        K-PKDPFLKKYCNPKKIQGLELQQIKTYGRQILEVLKFLHD---------
M_gi|20869393|ref|XP_127567.1|      K-PKDPFLKKYCNPKKTQGLELQQIKTYGRQILEALKFLHD---------
D_gi|21627748|gb|AAM68879.1|        ANPKNTFLSKYGNPKGRTALSMKQVATYGKQILEALIFLHS---------
D_gi|22945875|gb|AAN10635.1|AE      Q-WNEPWARKYT--RKPNGLPVSQVQRLGRQILEALLFLKE---------
D_gi|113194917|gb|AAF51744.3|       ----------------DLGLATLKNRSHAKSVIGTPEFMAP---------
H_gi|58530886|ref|NP_001561.3|      --------------GGSDPLPWPQRVSICSGLLCAVEYLHG---------
D_gi|21626698|gb|AAF47134.2|        ----------------RDYYTERDIAHYIRQTLWGLEHMHE---------
D_gi|17368346|sp|P83097|WSCK_D      LMAP---------SPRLTSLSEQLVLQWIYELASAMNYLSS---------
D_gi|21626698_cp                    -----------------HEYSEQMVATVVTQLLDALQYLHWR--------
M_gi|12839087|dbj|BAB24429.1|       ----------------YGACAETLAGKWFSQMALGIAYLHSKGIVHRLTP
H_gi|13027388|ref|NP_061041.2|      ---------------FPEGMSETLIRNILFGAVRGLNYLHQN--------
D_gi|17862032|gb|AAL39493.1|        --------------VYTSGFPEVAIALILKDVLSALTYIHS---------
H_gi|16306492|ref|NP_203698.1|      ---------------PGQYMDSSLVKVVTLWYRSPEVLLGS---------
D_gi|24641273                       ----------------MPNVTLHCLLDLMHGLVRGMHYLED---------
H_gi|34191428|gb|AAH36504.2|        --------------KAKKIIDSEWMQNVLGQVLDALEYLHH---------
H_gi|17368698|sp|Q9BXU1|STK31_      -----------------MPLNSEETLKVMKGVAQGLHTLHK---------
M_gi|20895826|ref|XP_139682.1|      -----------------QPLSNDHICYFLYQILQDLKYIHS---------
D_gi|21357711|ref|NP_647767.1|      ---------------NGPLLPEATIWSIIMQLTAGLKAIHHAG-------
                                                                          :           

D_gi|5052670|gb|AAD38665.1|AF1      SPPIIHGNLTCDSIFIQHNGLV----KIGSVVPDAVHYSVRRGRERERER
H_gi|14042287|dbj|BAB55185.1|       DPPIIHGNLTCDTIFIQHNGLI----KIGSVAPDTINNHVKTCREEQKN-
H_gi|22760645|dbj|BAC11278.1|       K--IVHRNLKLEN-LVYYNRLK----NSKIVISDFHLAKLENGLIKEPCG
H_gi|12052916|emb|CAB66632.1|       K--IVHRNLKLEN-LVYYNRLK----NSKIVISDFHLAKLENGLIKEPCG
M_gi|21704242|ref|NP_663596.1|      K--IVHRNLKLEN-LVYYNRLK----NSKIVISDFHLAKLENGLIKEPCG
R_gi|13027458|ref|NP_076490.1|      K--IVHRNLKLEN-LVYYNRLK----NSKIVISDFHLAKLENGLIKEPCG
H_gi|22760572|dbj|BAC11248.1|       K--IVHRNLKLEN-LVYYNRLK----NSKIVISDFHLAKLENGLIKEPCG
H_gi|4758292|ref|NP_004436.1|       FV---HRSLSAHSVLVN--------SHLVCKVARLGHSPQGPS-------
M_gi|21594381|gb|AAH31924.1|        FV---HRALSARSVLVN--------SHLVCKVARLGHSPQGSS-------
H_gi|119534|sp|P21860.1|ERBB3_      MV---HRNLAARNVLLK--------SPSQVQVADFGVADLLPPDDKQLLY
R_gi|149044565|gb|EDL97824.1|       LV---HGNVCTKNLLLAREGID-SDIGPFIKLSDPGIPVSVLTRQ-----
R_gi|109476840|ref|XP_00106164      LV---HGNVCTKNLLLAREGID-SDIGPFIKLSDPGIPVSVLTRQ-----
M_gi|111607496|ref|NP_666257.2      LV---HGNVCTKNLLLAREGID-SDIGPFIKLSDPGIPVSVLTRQ-----
R_gi|2288925|emb|CAA04187.1|        LV---HGNVCTKNLLLAREGID-SDIGPFIKLSDLGIPVSVLTRQ-----
H_ENSP00000294423                   LV---HGNVCTKNLLLAREGID-SECGPFIKLSDPGIPITVLSRQ-----
H_ENSP00000264818                   LV---HGNVCGRNILLARLGLA-EGTSPFIKLSDPGVGLGALSRE-----
M_gi|133922607|ref|NP_061263.2      LV---HGNVCGRNILLARLGLE-EGTNPFIKLSDPGVGQGALSRE-----
M_gi|156630890|sp|Q62137.2|JAK      LP---HGNVSARKVLLAREGG--DGNPPFIKLSDPGVSPTVLSLE-----
R_gi|2499671|sp|Q63272.1|JAK3_      LP---HGNVSARKVLLAREGV--DGNPPFIKLSDPGVSPTVLSLE-----
H_ENSP00000222246                   LP---HGNVSARKVLLAREGA--DGSPPFIKLSDPGVSPAVLSLE-----
M_gi|114326478|ref|NP_00104164      LI---HGNVCAKNILLIREEDRRTGNPPFIKLSDPGISITVLPKD-----
R_gi|2499669|sp|Q62689.1|JAK2_      LI---HGNVCAKNILLIREEDRKTGNPPFIKLSDPGISITVLPKD-----
H_ENSP00000371067                   LI---HGNVCAKNILLIREEDRKTGNPPFIKLSDPGISITVLPKD-----
D_gi|23093524|gb|AAN11824.1|AE      RF---HGALCTSNCLVDS--------RWVVKLTDFGLFAFKQGI-EDSS-
D_gi|7294467|gb|AAF49811.1|         RF---HGALCTSNCLVDS--------RWVVKLTDFGLFAFKQGI-EDSS-
D_gi|28380344|gb|AAF53079.3|        IS---HGNLRSSNCLIDS--------RWVCQISDFGLHELKAGQ-EEPNK
H_gi|40254426|ref|NP_000897.2|      CS---HGNLKSSNCVVDG--------RFVLKITDYGLESFR----DLDPE
R_gi|204270|gb|AAA41202.1|          CS---HGNLKSSNCVVDG--------RFVLKITDYGLESFR----DPEPE
R_gi|477540|pir||A49183             CS---HGNLKSSNCVVDG--------RFVLKITDYGLESFR----DPEPE
H_gi|4580422|ref|NP_003986.2|       SS---HGSLKSSNCVVDS--------RFVLKITDYGLASFRS---TAEPD
R_gi|16758694|ref|NP_446290.1|      SS---HGSLKSSNCVVDS--------RFVLKITDYGLASFRS---TAEPD
M_gi|20826487|ref|XP_131378.1|      SS---HGSLKSSNCVVDS--------RFVLKITDYGLASFRS---TAEPD
D_gi|23171402|gb|AAF55244.2|        RS---HGNLKSSNCVVDS--------RFVLKITDFGLHTLRRTRFDLESD
D_gi|7291217|gb|AAF46649.1|         AA---HGKLRSCNCLIDG--------RFVLKISDFGLRTLT---------
M_gi|159110415|ref|NP_032218.2      A----HGRLKSRNCVVDG--------RFVLKVTDHGHGRLLEAQRVLPEP
R_gi|13242283|ref|NP_077356.1|      A----HGRLKSRNCVVDG--------RFVLKVTDHGHGRLLEAQRVLPEP
H_gi|4504217|ref|NP_000171.1|       A----HGRLKSRNCIVDG--------RFVLKITDHGHGRLLEAQKVLPEP
R_gi|18543337|ref|NP_570093.1|      P----HGRLKSRNCVVDT--------RFVLKITDHGYAEFLESHCSFRPQ
M_gi|28529710|ref|XP_142224.2|      I----HGRLKSRNCVVDG--------RFVLKVTDYGFNDILEMLRLSEEE
M_gi|20349229|ref|XP_111982.1|      I----HGRLKSRNCVVDG--------RFVLKVTDYGFNDILEMLRLSEEE
R_gi|16758684|ref|NP_446283.1|      I----HGRLKSRNCVVDG--------RFVLKVTDYGFNNILEMLRLSEEE
H_gi|134152694|ref|NP_001513.2      V----HGRLKSRNCVVDG--------RFVLKVTDYGFNDILEMLRLSEEE
H_gi|18386335|gb|AAB19934.2|        EV---HGRLKSTNCVVDS--------RMVVKITDFGCNSILP--------
M_gi|21707860|gb|AAH34064.1|        EV---HGRLKSTNCVVDS--------RMVVKITDFGCNSILP--------
H_gi|4758606|ref|NP_004508.1|       IP---RHALNSRSVMIDE--------DMTARISMADVKFSFQCP------
R_gi|19173772|ref|NP_596900.1|      IP---RHALNSRSVMIDE--------DMTARISMADVKFSFQCP------
D_gi|24667933|ref|NP_525001.2|      IP---TYHLNSHHVMIDD--------DLTARINMGDAKFSFQEK------
H_gi|22749323|ref|NP_689862.1|      AP-ELHGKIRSSNFLVTQ--------GYQVKLAGFELRKTQTSMSLGT--
M_gi|12858445|dbj|BAB31320.1|       T---LHRNISSSSFLVAG--------GYQVKLAGFELSKTQNSISRTA--
H_gi|6005792|ref|NP_009130.1|       PCSVICGSISSANILLDD--------QFQPKLTDFAMAHFRSHLEHQS--
M_gi|28804246|emb|CAD29448.2|       PCAVICGNVSSANILLDD--------QLQPKLTDFAAAHFRPNLEQQS--
H_gi|57997202|emb|CAD38856.2|       F---IHRSLSSYAVHIIS--------PGEARLTNLEYMLESEDRGVQR--
M_gi|158635954|ref|NP_113563.2      F---IHRSLSSYAVHIVS--------AGEARLTNLEYLTESQDSGAHR--
D_gi|21358011|ref|NP_651655.1|      DAKIVHRNISAETIVINKNR--------SWKLFGFDFCIANQPATDGTPH
H_gi|7243101|dbj|BAA92598.1|        SVKMVHGNITPENIILNKSG--------AWKIMGFDFCVSSTNPSEQEPK
H_gi|14041796|dbj|BAB55454.1|       DCSLIHNNVCMAAVFVDRAG--------EWKLGGLDYMYSAQ----GNGG
H_gi|115430241|ref|NP_065731.3      DCSLIHNNVCMAAVFVDRAG--------EWKLGGLDYMYSAQ----GNGG
M_gi|12963867|ref|NP_076401.1|      DCNLIHNNVCMAAVFVDRAG--------EWKLGGLDYMYSAQ----GNGG
D_gi|7301824|gb|AAF56933.1|         DGNLRHNNVSAWSVFVNASG--------EWKLGSLEYVSAAD----GNP-
H_gi|15779207|gb|AAH14662.1|        RGHLTHNNVCLSSVFVSEDG--------HWKLGGMETVCKVS----QATP
M_gi|20829352|ref|XP_129532.1|      RGHLTHNNVCLSSVFVSEDG--------HWKLGGMETVCQVP----QATP
D_gi|45446806|gb|AAF45995.2|        RHHLVAHNVEPKHILLSSDGQ-------RVKLFNYGLHHMTKGGAYVPFP
H_gi|18676872|dbj|BAB85045.1|       KHGIVHRALSPHNILLDRKG--------HIKLAKFGLYHMTAHGDDVDFP
H_gi|31982929|ref|NP_057524.2|      --EYVHGNVTAENIFVDPED------QSQVTLAGYGFAFRYCPSGKHVAY
M_gi|21703091|gb|AAM74471.1|        --EYVHGNLTAENVFVNPED------LSQVTLVGYGFTYRYCPGGKHVAY
H_gi|7020363|dbj|BAA91097.1|        KGFPYG-HLHASNVMLDG---------DTCRLLDLEN-------------
M_gi|20869393|ref|XP_127567.1|      KGFPYG-HLHAANVMLDG---------NTCRLLDLEN-------------
D_gi|21627748|gb|AAM68879.1|        KGYAYG-HLHSGNIVIVD---------DCVKLLDIEN-------------
D_gi|22945875|gb|AAN10635.1|AE      RGFPLHGHLHSGNVILQN---------GAARLSGLEN-------------
D_gi|113194917|gb|AAF51744.3|       ------------------------------EMYEEHYDES----------
H_gi|58530886|ref|NP_001561.3|      -LEIIHSNVKSSNVLLDQN--------LTPKLAHPMAHLCPVNKRSK---
D_gi|21626698|gb|AAF47134.2|        -MGVGHMGLTIKDLLISVVG------GDIIKVSDFGLSRKINRHN-----
D_gi|17368346|sp|P83097|WSCK_D      -CQVVHRQLCSHSVFVTS--------DFKLKLSVFGPLPYMNIAR-----
D_gi|21626698_cp                    --GYCHLNIQPDNVVMASVR------SIQVKLVDFGSAKKVNKLGMKVTP
M_gi|12839087|dbj|BAB24429.1|       SLSAAGRDLKLENLLLDK--------RENVKISDFGFAKMVPSSQPVHSS
H_gi|13027388|ref|NP_061041.2|      --GCIHRSIKASHILISGDG--------LVTLSGLSHLHSLVKHGQRHRA
D_gi|17862032|gb|AAL39493.1|        -EHYVHGSVRAKHILLSP---------RKAVLSNFSYCQSFISQGEKKTF
H_gi|16306492|ref|NP_203698.1|      -----ARYSTPVDIWSIG-----------TIFAELATKKPLFHGD-----
D_gi|24641273                       -NKIIHNYIRCSNLYVTKYDPN--SYVLDAKISDPGYPRPYRE-------
H_gi|34191428|gb|AAH36504.2|        -LDIIHRNLKPSNIILIS--------SDHCKLQDLSSNVLMTDKAKWNIR
H_gi|17368698|sp|Q9BXU1|STK31_      -ADIIHGSLHQNNVFALN--------REQGIVGDFDFTKSVSQRASVN--
M_gi|20895826|ref|XP_139682.1|      --------ANTCDFGLAR-------------IADPEHDHTG---------
D_gi|21357711|ref|NP_647767.1|      ---LACKVLDPTKIIVTG---------KRVRFSSCCISDITQFDP-----
                                                                                      

D_gi|5052670|gb|AAD38665.1|AF1      E---------RGAHYFQAPEYGAADQLTAA-------LDIYAFG------
H_gi|14042287|dbj|BAB55185.1|       -------------LHFFAPEYGEVTNVTTA-------VDIYSFG------
H_gi|22760645|dbj|BAC11278.1|       ------------TPEYLAPEVVGRQRYGRP-------VDCWAIGVIMYIL
H_gi|12052916|emb|CAB66632.1|       ------------TPEYLG--------------------------------
M_gi|21704242|ref|NP_663596.1|      ------------TPEYLAPEVVGRQRYGRP-------VDCWAIGVIMYIL
R_gi|13027458|ref|NP_076490.1|      ------------TPEYLAPEVVGRQRYGRP-------VDCWAIGVIMYIL
H_gi|22760572|dbj|BAC11248.1|       ------------TPEYLAPEVVGRQRYGRP-------VDCWAIGVIMYIL
H_gi|4758292|ref|NP_004436.1|       -----------CLLRWAAPEVIAH--GKHT-----TSSDVWSFGILMWEV
M_gi|21594381|gb|AAH31924.1|        -----------SLLRWAAPEVITH--GKYT-----TSSDVWSFGILMWEV
H_gi|119534|sp|P21860.1|ERBB3_      -------SEAKTPIKWMALESIHF--GKYT-----HQSDVWSYGVTVWEL
R_gi|149044565|gb|EDL97824.1|       --------ECIERIPWIAPECVED-SKNLS-----VAADKWSFGTTLWEI
R_gi|109476840|ref|XP_00106164      --------ECIERIPWIAPECVED-SKNLS-----VAADKWSFGTTLWEI
M_gi|111607496|ref|NP_666257.2      --------ECIERIPWIAPECVED-SKNLS-----VAADKWSFGTTLWEI
R_gi|2288925|emb|CAA04187.1|        --------ECIERNPWIAPECVED-SKNLS-----VAADKWSFGTTLWEI
H_ENSP00000294423                   --------ECIERIPWIAPECVED-SKNLS-----VAADKWSFGTTLWEI
H_ENSP00000264818                   --------ERVERIPWLAPECLPGGANSLS-----TAMDKWGFGATLLEI
M_gi|133922607|ref|NP_061263.2      --------ERVERIPWTAPECLSGGTSSLG-----TATDMWGFGATLLEI
M_gi|156630890|sp|Q62137.2|JAK      --------MLTDRIPWVAPECLQE-AQTLC-----LEADKWGFGATTWEV
R_gi|2499671|sp|Q63272.1|JAK3_      --------MLTDRIPWVAPECLQE-AGTLN-----LEADKWGFGATTWEV
H_ENSP00000222246                   --------MLTDRIPWVAPECLRE-AQTLS-----LEADKWGFGATVWEV
M_gi|114326478|ref|NP_00104164      --------ILQERIPWVPPECIEN-PKNLN-----LATDKWSFGTTLWEI
R_gi|2499669|sp|Q62689.1|JAK2_      --------ILQERIPWVPPECIEN-PKNLT-----LATDKWSFGTTLWEI
H_ENSP00000371067                   --------ILQERIPWVPPECIEN-PKNLN-----LATDKWSFGTTLWEI
D_gi|23093524|gb|AAN11824.1|AE      TDMQHM-SAKCLKLLYRAPELLRQGPSSLV--MGTQRGDAYSFGILLYEM
D_gi|7294467|gb|AAF49811.1|         TDMQHM-SAKCLKLLYRAPELLRQGPSSLV--MGTQRGDAYSFGILLYEM
D_gi|28380344|gb|AAF53079.3|        SELELK-RALCM-----APELLR-DAYRPG--RGSQKGDVYSFGILLYEM
H_gi|40254426|ref|NP_000897.2|      ----QG-HTVYAKKLWTAPELLRMAS-PPV--RGSQAGDVYSFGIILQEI
R_gi|204270|gb|AAA41202.1|          ----QG-HTLFAKKLWTAPELLRMAS-PPA--RGSQAGDVYSFGIILQEI
R_gi|477540|pir||A49183             ----QG-HTLFAKKLWTAPELLRMAS-PPA--RGSQAGDVYSFGIILQEI
H_gi|4580422|ref|NP_003986.2|       ----DS-HALYAKKLWTAPELLSGNP-LPT--TGMQKADVYSFGIILQEI
R_gi|16758694|ref|NP_446290.1|      ----DS-HALYAKKLWTAPELLSGNP-LPT--TGMQKADVYSFAIILQEI
M_gi|20826487|ref|XP_131378.1|      ----DS-HALYAKKLWTAPELLSGNP-LPT--TGMQKADVYSFAIILQEI
D_gi|23171402|gb|AAF55244.2|        GGNCNS-HAYWSKLLWTAPELLRVEHNRPP--EGTQKGDVYAFGIIVHEI
D_gi|7291217|gb|AAF46649.1|         ----------------TPSDFVRDQN--YY--LTTQRGDVYSFGIILEEI
M_gi|159110415|ref|NP_032218.2      --------PSAEDQLWTAPELLRDPSLER---RGTLAGDVFSLAIIMQEV
R_gi|13242283|ref|NP_077356.1|      --------PSAEDQLWTAPELLRDPALER---RGTLAGDVFSLGIIMQEV
H_gi|4504217|ref|NP_000171.1|       --------PRAEDQLWTAPELLRDPALER---RGTLAGDVFSLAIIMQEV
R_gi|18543337|ref|NP_570093.1|      --------PAPEELLWTAPELLRGPRGPWGPGKATFKGDVFSLGIILQEV
M_gi|28529710|ref|XP_142224.2|      --------PSEEELLWTAPELLRAPGGIR---LGSFAGDVYSFAIIMQEV
M_gi|20349229|ref|XP_111982.1|      --------PSEEELLWTAPELLRAPGGIR---LGSFAGDVYSFAIIMQEV
R_gi|16758684|ref|NP_446283.1|      --------PSEEELLWTAPELLRAPGGIR---LGSFAGDVYSFAIIMQEV
H_gi|134152694|ref|NP_001513.2      --------SSMEELLWTAPELLRAPRGSR---LGSFAGDVYSFAIIMQEV
H_gi|18386335|gb|AAB19934.2|        ----------PKKDLWTAPEHLRQAN-------ISQKGDVYSYGIIAQEI
M_gi|21707860|gb|AAH34064.1|        ----------PKKDLWTAPEHLRQAT-------ISQKGDVYSFAIIAQEI
H_gi|4758606|ref|NP_004508.1|       --------GRMYAPAWVAPEALQKKPEDTN----RRSADMWSFAVLLWEL
R_gi|19173772|ref|NP_596900.1|      --------GRMYAPAWVAPEALQKKPEDTN----RRSADMWSFAVLLWEL
D_gi|24667933|ref|NP_525001.2|      --------GRIYQPAWMSPETLQRKQADRN----WEACDMWSFAILIWEL
H_gi|22749323|ref|NP_689862.1|      --------TREKTDRVKSTAYLSPQELEDVFYQYDVKSEIYSFGIVLWEI
M_gi|12858445|dbj|BAB31320.1|       --------KSTKAERSSSTIYVSPERLKNPFCLYDIKAEIYSFGIVLWEI
H_gi|6005792|ref|NP_009130.1|       --------CTINMTSSSSKHLWYMPEEYIRQGKLSIKTDVYSFGIVIMEV
M_gi|28804246|emb|CAD29448.2|       --------STINMTGGGRKHLWYMPEEYIRQGRLSVKTDVYSFGIVIMEV
H_gi|57997202|emb|CAD38856.2|       --------DLTRVPLPTQLYNWAAPEVILQK-AATVKSDIYSFSMIMQEI
M_gi|158635954|ref|NP_113563.2      --------NVTRMPLPTQLYNWAAPEVVLQK-AATVKSDIYSFSVIIQEI
D_gi|21358011|ref|NP_651655.1|      WPFRE---YTTSLHVLAQPSLEYTAPELALNSVNTPDSDLFSLGVLIFTI
H_gi|7243101|dbj|BAA92598.1|        FPCKE---WDPNLPSLCLPNPEYLAPEYILSVSCETASDMYSLGTVMYAV
H_gi|14041796|dbj|BAB55454.1|       GPPRK---GIPELEQYDPPELADSS---GRVVREKWSADMWRLGCLIWEV
H_gi|115430241|ref|NP_065731.3      GPPRK---GIPELEQYDPPELADSS---GRVVREKWSADMWRLGCLIWEV
M_gi|12963867|ref|NP_076401.1|      GPPSK---GIPELEQYDPPELADSS---SRAVREKWSADMWRLGCLIWEV
D_gi|7301824|gb|AAF56933.1|         MPPAK---IPVTLEVYDSPEKNDPS---KLKAATKCSVDMWGLGCLVWEA
H_gi|15779207|gb|AAH14662.1|        EFLRS---IQSIRDPASIPPEEMSP---EFTTLPECHGHAR-DAFSFGTL
M_gi|20829352|ref|XP_129532.1|      EFLRN---IQSVRDPASIPPEEMSP---EFSGLPESHGHAR-DAYAFGAL
D_gi|45446806|gb|AAF45995.2|        IGNIR---YMAPERLLG--------------LNGNVKSDVWSLALVMVEL
H_gi|18676872|dbj|BAB85045.1|       IGYPS---YLAPEVIAQGIFKTTDHMPSKKPLPSGPKSDVWSLGIILFEL
H_gi|31982929|ref|NP_057524.2|      V-------EGSRSPHEGDLEFISMDLHKGCG--PSRRSDLQSLGYCMLKW
M_gi|21703091|gb|AAM74471.1|        K-------EGSRSPHDGDLEFISMDLHKGCG--PSRRSDLQTLGYCMLKW
H_gi|7020363|dbj|BAA91097.1|        -------------SLLGLPSFYRSYFSQFRKINTLESVDVHCFGHLLYEM
M_gi|20869393|ref|XP_127567.1|      -------------SLLGLPSFYRSYFTQFRKINTLESVDVHCFGHLLYEM
D_gi|21627748|gb|AAM68879.1|        -------------FLLGVPAFYRPFFMQHSKIHAIETIDVYCFGHVLFEM
D_gi|22945875|gb|AAN10635.1|AE      -------------GLLGLSSRIN-AVMWSRSVTEIENVDIVCFGHLLYEM
D_gi|113194917|gb|AAF51744.3|       -------------------------------------VDVYAFGMCMLEM
H_gi|58530886|ref|NP_001561.3|      --------YTMMKTHLLRTSAAYLPEDFIRVGQLTKRVDIFSCGIVLAEV
D_gi|21626698|gb|AAF47134.2|        ----------LSTLDYGMPEFVSPEVVNKE--GVNFSHDMWTVGLITYVL
D_gi|17368346|sp|P83097|WSCK_D      --------QQPDHNRWLAPEVLRHQHHHST------RSDVWSLACVAWEC
D_gi|21626698_cp                    ----------CGSLDFQPPEMINDEPIFPQ-------SDIWSLGALTYLL
M_gi|12839087|dbj|BAB24429.1|       PSYRQMNSLSHLSQTYCGSFAYACPEILLGLPYNPFLSDTWSMGVILYTL
H_gi|13027388|ref|NP_061041.2|      VYDFP--QFSTSVQPWLSPELLRQDLHGYN-----VKSDIYSVGITACEL
D_gi|17862032|gb|AAL39493.1|        IFG--STVGIEKELYWTAPEVLYQNLSGYT-----EKIDIYSIGITCCEM
H_gi|16306492|ref|NP_203698.1|      ----------------SEIDQLFRIFRALG----TPNNEVWPEVESLQDY
D_gi|24641273                       -----------SDSPWIPVKYYRNLQAAKT----DQFAQLWAFATTIYEI
H_gi|34191428|gb|AAH36504.2|        AE------EDPFRKSWMAPEALNFSFSQKS--------DIWSLGCIILDM
H_gi|17368698|sp|Q9BXU1|STK31_      --------MMVGDLSLMSPELKMGKPASPG-------SDLYAYGCLLLWL
M_gi|20895826|ref|XP_139682.1|      ---------FLTRWPRTAPQIVLNSKSYPK------SIDFWSLGCVLAEL
D_gi|21357711|ref|NP_647767.1|      ----------NASNPLALVNMHQQ-------------DDLTALGRLVLAL
                                                                                      

D_gi|5052670|gb|AAD38665.1|AF1      -------MCALE----------------------MAALEIQPSNSES---
H_gi|14042287|dbj|BAB55185.1|       -------MCALE----------------------MAVLEIQ-GNGES---
H_gi|22760645|dbj|BAC11278.1|       LSGNPPFYEEVE----------------------EDDYENHDKNLFR---
H_gi|12052916|emb|CAB66632.1|       ---NPPFYEEVE----------------------EDDYENHDKNLFR---
M_gi|21704242|ref|NP_663596.1|      LSGNPPFYEEVE----------------------EDDYENHDKNLFR---
R_gi|13027458|ref|NP_076490.1|      LSGNPPFYEEVE----------------------EDDYENHDKNLFR---
H_gi|22760572|dbj|BAC11248.1|       LSGNPPFYEEVE----------------------EDDYENHDKNLFR---
H_gi|4758292|ref|NP_004436.1|       MSYGERPYWDM------------------------SEQEVLNAIEQ----
M_gi|21594381|gb|AAH31924.1|        MSYGERPYWDM------------------------NEQEVLNAIEQ----
H_gi|119534|sp|P21860.1|ERBB3_      MTFGAEPYAGL------------------------RLAEVPDLLEK----
R_gi|149044565|gb|EDL97824.1|       CYNGEIPLKDK------------------------TLIEKERFYES----
R_gi|109476840|ref|XP_00106164      CYNGEIPLKDK------------------------TLIEKERFYES----
M_gi|111607496|ref|NP_666257.2      CYNGEIPLKDK------------------------TLIEKERFYES----
R_gi|2288925|emb|CAA04187.1|        RYDGEIPLKDK------------------------TLIEKERFYES----
H_ENSP00000294423                   CYNGEIPLKDK------------------------TLIEKERFYES----
H_ENSP00000264818                   CFDGEAPLQSR------------------------SPSEKEHFYQR----
M_gi|133922607|ref|NP_061263.2      CFDGEAPLQGR------------------------GPSEKERFYTK----
M_gi|156630890|sp|Q62137.2|JAK      FSGGPAHITSL------------------------EPAKKLKFYED----
R_gi|2499671|sp|Q63272.1|JAK3_      FSGAPMHITSL------------------------EPAKKLKFYED----
H_ENSP00000222246                   FSGVTMPISAL------------------------DPAKKLQFYED----
M_gi|114326478|ref|NP_00104164      CSGGDKPLSAL------------------------DSQRKLQFYED----
R_gi|2499669|sp|Q62689.1|JAK2_      CSGGDKPLSAL------------------------DSQRKLQFYED----
H_ENSP00000371067                   CSGGDKPLSAL------------------------DSQRKLQFYED----
D_gi|23093524|gb|AAN11824.1|AE      HVRRGPFGETG-----------------------LTPMQCLQKVLQ----
D_gi|7294467|gb|AAF49811.1|         HVRRGPFGETG-----------------------LTPMQCLQKVLQ----
D_gi|28380344|gb|AAF53079.3|        IGRKGPWGDTA-----------------------YSKEEIIQFVKC----
H_gi|40254426|ref|NP_000897.2|      ALRSGVFHVEG---------------------LDLSPKEIIERVT-----
R_gi|204270|gb|AAA41202.1|          ALRSGVFYVEG---------------------LDLSPKEIIERVT-----
R_gi|477540|pir||A49183             ALRSGVFYVEG---------------------LDLSPKEIIERVT-----
H_gi|4580422|ref|NP_003986.2|       ALRSGPFYLEG---------------------LDLSPKEIVQKVR-----
R_gi|16758694|ref|NP_446290.1|      ALRSGPFYLEG---------------------LDLSPKEIVQKVR-----
M_gi|20826487|ref|XP_131378.1|      ALRSGPFYLEG---------------------LDLSPKEIVQKVR-----
D_gi|23171402|gb|AAF55244.2|        TTRQGPFYLGRC-------------------AYEKSPQEIIELVKGY---
D_gi|7291217|gb|AAF46649.1|         VNRGGPYQEAR---------------------QQMDVHTILHKVR-----
M_gi|159110415|ref|NP_032218.2      VCRSTPYAMLE-----------------------LTPEEVIQRVRS----
R_gi|13242283|ref|NP_077356.1|      VCRSTPYAMLE-----------------------LTPEEVIQRVRS----
H_gi|4504217|ref|NP_000171.1|       VCRSAPYAMLE-----------------------LTPEEVVQRVRS----
R_gi|18543337|ref|NP_570093.1|      LTRDPPYCSWG-----------------------LSAEEIIRKVAS----
M_gi|28529710|ref|XP_142224.2|      MVRGAPFCMMD-----------------------LPAKEIIDRLKM----
M_gi|20349229|ref|XP_111982.1|      MVRGAPFCMMD-----------------------LPAKEIIDRLKM----
R_gi|16758684|ref|NP_446283.1|      MVRGAPFCMMD-----------------------LSAKEVIDRLKM----
H_gi|134152694|ref|NP_001513.2      MVRGTPFCMMD-----------------------LPAQEIINRLKK----
H_gi|18386335|gb|AAB19934.2|        ILRKETFYTLS----------------------CRDRNEKIFRVEN----
M_gi|21707860|gb|AAH34064.1|        ILRKETFYTLS----------------------CRDHNEKIFRVEN----
H_gi|4758606|ref|NP_004508.1|       VTREVPFADLS-------------------------NMEIGMKVAL----
R_gi|19173772|ref|NP_596900.1|      VTREVPFADLS-------------------------NMEIGMKVAL----
D_gi|24667933|ref|NP_525001.2|      TTREVPFAEWS-------------------------PMECGMKIAL----
H_gi|22749323|ref|NP_689862.1|      ATG-DIPFQGC------------------------NSEKIRKLVAV----
M_gi|12858445|dbj|BAB31320.1|       ATG-KIPFEGC------------------------DSKKIRELVAE----
H_gi|6005792|ref|NP_009130.1|       LTGCRVVLDDP------------------------KHIQLRDLLRELMEK
M_gi|28804246|emb|CAD29448.2|       LTGCKVVLDDP------------------------KHVQLRDLLMELMEK
H_gi|57997202|emb|CAD38856.2|       LTD-DIPWKGL------------------------DGSVVKKAVVSG---
M_gi|158635954|ref|NP_113563.2      LTD-SIPWNGL------------------------DGSLVKETIALG---
D_gi|21358011|ref|NP_651655.1|      Y-AGKPLKMFGS-------------------D--YSSFRRYAN-------
H_gi|7243101|dbj|BAA92598.1|        FNKGKPIFEVNK-------------------QDIYKSFSRQLD-------
H_gi|14041796|dbj|BAB55454.1|       FNGPLPRAAALR-------------------N------------------
H_gi|115430241|ref|NP_065731.3      FNGPLPRAAALR-------------------N------------------
M_gi|12963867|ref|NP_076401.1|      FNGSLPRAAALR-------------------N------------------
D_gi|7301824|gb|AAF56933.1|         FNGVLKQRSNLK-------------------D------------------
H_gi|15779207|gb|AAH14662.1|        VESLLTILNEQV-------------------S------------------
M_gi|20829352|ref|XP_129532.1|      VDSLLPIFNEQV-------------------S------------------
D_gi|45446806|gb|AAF45995.2|        ILQIELWPKLKL-------------------SNVVRKILAFGKSNGA---
H_gi|18676872|dbj|BAB85045.1|       CVGRKLFQSLDI-------------------SERLKFLLTLDCVDDT---
H_gi|31982929|ref|NP_057524.2|      LYGFLPWTNCLP-----------------------NTEDIMKQKQK----
M_gi|21703091|gb|AAM74471.1|        LYGSLPWTNCLP-----------------------NTEKITRQKQK----
H_gi|7020363|dbj|BAA91097.1|        TYGRPPDSVPVD-------------------SFPPAPSMAVVAVLES---
M_gi|20869393|ref|XP_127567.1|      TYGRPPDSVPVD-------------------SFPPASSLAVVAVLES---
D_gi|21627748|gb|AAM68879.1|        AMGYPLQESVVR-------------------QITECP-EALKCLLES---
D_gi|22945875|gb|AAN10635.1|AE      CTG-----------------------------------------------
D_gi|113194917|gb|AAF51744.3|       AISEYPYSECKG------------------------PAQIYKKVISG---
H_gi|58530886|ref|NP_001561.3|      LTGIPAMDNNRS-------------------PVYLKDLLLSDIPSSTASL
D_gi|21626698|gb|AAF47134.2|        LGGHNPFLGID-------------------------DRETLTKIREG---
D_gi|17368346|sp|P83097|WSCK_D      CALGGTPYANAV-------------------ASNQQLLEAIRAAVR----
D_gi|21626698_cp                    LSGCSPFRGADE-------------------YETKQNISFVRYRFEN---
M_gi|12839087|dbj|BAB24429.1|       VVARLPFDDTN-------------------------LKKLLRETQK----
H_gi|13027388|ref|NP_061041.2|      ASGQVPFQDMHRTQMLLQKLKGPPYSPLDISIFPQSESRMKNSQSGVDSG
D_gi|17862032|gb|AAL39493.1|        ANGFQPFKDTELTYMYIEKVRG-------SLQVLLDKNSLLENQGSLS--
H_gi|16306492|ref|NP_203698.1|      KNTFPKWKPGS----------------------------LASHVKN----
D_gi|24641273                       FSRCKEDLSTLR----------------------QEQLLRQKNLDGN---
H_gi|34191428|gb|AAH36504.2|        TSCSFMDGTEAM-----------------------HLRKSLRQSPGS---
H_gi|17368698|sp|Q9BXU1|STK31_      SVQNQEFEINKD-----------------------GIPKVDQFHLD----
M_gi|20895826|ref|XP_139682.1|      LSNQPTIFPGQH--------------------YLTQPNHILDYYKMN---
D_gi|21357711|ref|NP_647767.1|      ACRCLQSVQRDN------------------------VQSSIDMVTRN---
                                                                                      

D_gi|5052670|gb|AAD38665.1|AF1      ----------TAIN-EETIQRTIFSLENDLQRDLIRKCLNPQPQDRPSAN
H_gi|14042287|dbj|BAB55185.1|       ----------SYVP-QEAISSAIQLLEDPLQREFIQKCLQSEPARRPTAR
H_gi|22760645|dbj|BAC11278.1|       ----------KILAGDYEFDSPYWDDISQAAKDLVTRLMEVEQDQRITAE
H_gi|12052916|emb|CAB66632.1|       ----------KILAGDYEFDSPYWDDISQAAKDLVTRLMEVEQDQRITAE
M_gi|21704242|ref|NP_663596.1|      ----------KILAGDYEFDSPYWDDISQAAKDLVTRLMEVEQDQRITAE
R_gi|13027458|ref|NP_076490.1|      ----------KILAGDYEFDSPYWDDISQAAKDLVTRLMEVEQDQRITAE
H_gi|22760572|dbj|BAC11248.1|       ----------KILAGDYEFDSPYWDDISQAAKDLVTRLMEVEQDQRITAE
H_gi|4758292|ref|NP_004436.1|       ----------EFRLPPPPGC-------PPGLHLLMLDTWQKDRARRPHFD
M_gi|21594381|gb|AAH31924.1|        ----------EFRLPPPPGC-------PPGLHLLMLDTWQKDRARRPHFD
H_gi|119534|sp|P21860.1|ERBB3_      ----------GERLAQPQIC-------TIDVYMVMVKCWMIDENIRPTFK
R_gi|149044565|gb|EDL97824.1|       ----------RCRPVTPS---------CKELADLMTRCMNYDPNQRPFFR
R_gi|109476840|ref|XP_00106164      ----------RCRPVTPS---------CKELADLMTRCMNYDPNQRPFFR
M_gi|111607496|ref|NP_666257.2      ----------RCRPVTPS---------CKELADLMTRCMNYDPNQRPFFR
R_gi|2288925|emb|CAA04187.1|        ----------RCRPVTPS---------CKELADLMTRCMNYDPNQRPFFR
H_ENSP00000294423                   ----------RCRPVTPS---------CKELADLMTRCMNYDPNQRPFFR
H_ENSP00000264818                   ----------QHRLPEPS---------CPQLATLTSQCLTYEPTQRPSFR
M_gi|133922607|ref|NP_061263.2      ----------KHQLPEPS---------SPELATLTRQCLTYEPAQRPSFR
M_gi|156630890|sp|Q62137.2|JAK      ----------QGQLPALK---------WTELAGLITQCMAYDPGRRPSFR
R_gi|2499671|sp|Q63272.1|JAK3_      ----------RGQLPALK---------WTELEGLIAQCMAYDPGRRPSFR
H_ENSP00000222246                   ----------RQQLPAPK---------WTELALLIQQCMAYEPVQRPSFR
M_gi|114326478|ref|NP_00104164      ----------KHQLPAPK---------WTELANLINNCMDYEPDFRPAFR
R_gi|2499669|sp|Q62689.1|JAK2_      ----------KHQLPAPK---------WTELANLINTCMDYEPDFRPAFR
H_ENSP00000371067                   ----------RHQLPAPK---------WAELANLINNCMDYEPDFRPSFR
D_gi|23093524|gb|AAN11824.1|AE      ------PQDYLN-PYRPSLQPLET-AF-DCVSECLRECWAERPEDRPDFK
D_gi|7294467|gb|AAF49811.1|         ------PQDYLN-PYRPSLQPLET-AF-DCVSECLRECWAERPEDRPDFK
D_gi|28380344|gb|AAF53079.3|        ------PEMLQHGVFRPALTHTHL-DIPDYIRKCLCQCWDEDPEVRPDIR
H_gi|40254426|ref|NP_000897.2|      --------RGEQPPFRPSLA-LQ--SHLEELGLLMQRCWAEDPQERPPFQ
R_gi|204270|gb|AAA41202.1|          --------RGEQPPFRPSMD-LQ--SHLEELGQLMQRCWAEDPQERPPFQ
R_gi|477540|pir||A49183             --------RGEQPPFRPSMD-LQ--SHLEELGQLMQRCWAEDPQERPPFQ
H_gi|4580422|ref|NP_003986.2|       --------NGQRPYFRPSID-RT--QLNEELVLLMERCWAQDPAERPDFG
R_gi|16758694|ref|NP_446290.1|      --------NGQRPYFRPSID-RT--QLNEELVLLMERCWAQDPTERPDFG
M_gi|20826487|ref|XP_131378.1|      --------NGQRPYFRPSID-RT--QLNEELVLLMERCWAQDPTERPDFG
D_gi|23171402|gb|AAF55244.2|        ------NPHRMQKPFRPELE-PNG-DTKADINGIIRRCWAEDPAERPDFN
D_gi|7291217|gb|AAF46649.1|         ----------QCNGFRPLIR-ER--ECPPDLLELMEKCWADNQEERPTFS
M_gi|159110415|ref|NP_032218.2      ----------PPPLCRPLVS--MD-QAPMECIQLMTQCWAEHPELRPSMD
R_gi|13242283|ref|NP_077356.1|      ----------PPPLCRPLVS--MD-QAPMECIQLMAQCWAEHPELRPSMD
H_gi|4504217|ref|NP_000171.1|       ----------PPPLCRPLVS--MD-QAPVECILLMKQCWAEQPELRPSMD
R_gi|18543337|ref|NP_570093.1|      ----------PPPLCRPLVS--PD-QGPLECIQLMQLCWEEAPDDRPSLD
M_gi|28529710|ref|XP_142224.2|      ----------PPPVYRPVVS--PE-YAPAECLQLMKQCWAEASEQRPTFD
M_gi|20349229|ref|XP_111982.1|      ----------PPPVYRPVVS--PE-YAPAECLQLMKQCWAEASEQRPTFD
R_gi|16758684|ref|NP_446283.1|      ----------PPPVYRPVVS--PE-FAPPECLQLMKQCWAEAAEQRPTFD
H_gi|134152694|ref|NP_001513.2      ----------PPPVYRPVVP--PE-HAPPECLQLMKQCWAEAAEQRPTFD
H_gi|18386335|gb|AAB19934.2|        --------SNGMKPFRPDLFLETAEEKELEVYLLVKNCWEEDPEKRPDFK
M_gi|21707860|gb|AAH34064.1|        --------SYG-KPFRPDLFLETADEKELEVYLLVKSCWEEDPEKRPDFK
H_gi|4758606|ref|NP_004508.1|       ------------EGLRPTIP----PGISPHVCKLMKICMNEDPAKRPKFD
R_gi|19173772|ref|NP_596900.1|      ------------EGLRPTIP----PGISPHVCKLMKICMNEDPAKRPKFD
D_gi|24667933|ref|NP_525001.2|      ------------EGLRVKIP----PGTSTHMAKLISICMNEDPGKRPKFD
H_gi|22749323|ref|NP_689862.1|      ----------KRQQEPLGED------CPSELREIIDECRAHDPSVRPSVD
M_gi|12858445|dbj|BAB31320.1|       ----------DKKQEPVGQD------CPELLREIINECRAHEPSQRPSVD
H_gi|6005792|ref|NP_009130.1|       RGLDSCLSFLDKKVPPCPRN------FSAKLFCLAGRCAATRAKLRPSMD
M_gi|28804246|emb|CAD29448.2|       RGLDSCLSFLDRKIPPCPRN------FSAKLFSLAGRCVATKAKLRPTMD
H_gi|57997202|emb|CAD38856.2|       ------------NYLEADVR------LPKPYYDIVKSGIHVKQKDR-TMN
M_gi|158635954|ref|NP_113563.2      ------------NYLEADVR------LPEPYYDIVKSGIHAKQKNR-TMN
D_gi|21358011|ref|NP_651655.1|      --------------DLNQRKYPPMNAVPSELTESLKALLHPSANLRPKLH
H_gi|7243101|dbj|BAA92598.1|        --------------QLSRLGSSSLTNIPEEVREHVKLLLNVTPTVRPDAD
H_gi|14041796|dbj|BAB55454.1|       -----------------------PGKIPKTLVPHYCELVGANPKVRPNPA
H_gi|115430241|ref|NP_065731.3      -----------------------PGKIPKTLVPHYCELVGANPKVRPNPA
M_gi|12963867|ref|NP_076401.1|      -----------------------PGKIPKSLVTHYCELVGANPKVRPNPA
D_gi|7301824|gb|AAF56933.1|         -----------------------IEHIPKSLQSLYCELVGASPSNRPNPA
H_gi|15779207|gb|AAH14662.1|        -----------------------ADVLSSFQQTLHSTLLNPIPKCRPALC
M_gi|20829352|ref|XP_129532.1|      -----------------------ADVLSSFLQILHSALLNPMPECRPALS
D_gi|45446806|gb|AAF45995.2|        ----------LEKIAREHQCHERYVQMDQRLRQLLESCLSVLPKRRPLPG
H_gi|18676872|dbj|BAB85045.1|       ----------LIVLAEEHGCLDIIKELPETVIDLLNKCLTFHPSKRPTPD
H_gi|31982929|ref|NP_057524.2|      ---------FVDKPGPFVGPCGHWIRPSETLQKYLKVVMALTYEEKPPYA
M_gi|21703091|gb|AAM74471.1|        ---------YLDSPERLVGLCGRWNKASETLREYLKVVMALNYEEKPPYA
H_gi|7020363|dbj|BAA91097.1|        --------TLSCEACKNG---------MPTISRLLQMPLFSDVLLTTSEK
M_gi|20869393|ref|XP_127567.1|      --------TLSCEACKNG---------MPTVSRLLQMPLFSDVLLTTSEK
D_gi|21627748|gb|AAM68879.1|        --------ILSKEACKAG---------LPTLEQLLGHRFF----------
D_gi|22945875|gb|AAN10635.1|AE      ------------QELTTP---------KPSMRVL----------------
D_gi|113194917|gb|AAF51744.3|       ----------IKPAALAKVE-------DPNVRDIIERCIELKKEDRPSCN
H_gi|58530886|ref|NP_001561.3|      CSRKTGVENVMAKEICQKYLEKGAGRLPEDCAEALATAACLCLRRRNTSL
D_gi|21626698|gb|AAF47134.2|        ---------------RWDFKDEIWTHISDDGRDFISRLLLYSPEERMDVK
D_gi|17368346|sp|P83097|WSCK_D      -------------PAQPAYV-------YGDLYQLLLNCWQLEPSERSSCE
D_gi|21626698_cp                    ----------------------LFKEVTPEATRFIMLLFKRHPTKRPYTE
M_gi|12839087|dbj|BAB24429.1|       ------------------------EVTFPANLTISQECKGSSIKPGPQPL
H_gi|13027388|ref|NP_061041.2|      IGESVLVSSGTHTVNSDRLHTPSSKTFSPAFFSLVQLCLQQDPEKRPSAS
D_gi|17862032|gb|AAL39493.1|        ---------LEHTNKRIARDVIVNKSFSENFHQFVELCLNKNPLSRWAAS
H_gi|16306492|ref|NP_203698.1|      --------------------------LDENGLDLLSKMLIYDPAKRISGK
D_gi|24641273                       ----------ILKMLDQDIC-------PAPIFETIMDGWSDDETKRFSHH
H_gi|34191428|gb|AAH36504.2|        ------------LKAVLKTMEEKQIPDVETFRNLLPLMLQIDPSDRITIK
H_gi|17368698|sp|Q9BXU1|STK31_      ----------------------------DKVKSLLCSLICYRSSMTAEQV
M_gi|20895826|ref|XP_139682.1|      ----------FAQHKPQKPRLDQGWSWPPSLTLLGSPTSSRLLPLQSPAP
D_gi|21357711|ref|NP_647767.1|      --------------------------YSTDLRNFIVYLFTTNNRRSVTDL
                                                                                      

D_gi|5052670|gb|AAD38665.1|AF1      -------DLLFHPLL-----------------------------------
H_gi|14042287|dbj|BAB55185.1|       -------ELLFHPAL-----------------------------------
H_gi|22760645|dbj|BAC11278.1|       -------EAISHEWI-----------------------------------
H_gi|12052916|emb|CAB66632.1|       -------EAISHEWI-----------------------------------
M_gi|21704242|ref|NP_663596.1|      -------EAISHEWI-----------------------------------
R_gi|13027458|ref|NP_076490.1|      -------EAISHEWI-----------------------------------
H_gi|22760572|dbj|BAC11248.1|       -------EAISHESI-----------------------------------
H_gi|4758292|ref|NP_004436.1|       --------QLVAAF------------------------------------
M_gi|21594381|gb|AAH31924.1|        --------QLVAAF------------------------------------
H_gi|119534|sp|P21860.1|ERBB3_      --------ELANEF------------------------------------
R_gi|149044565|gb|EDL97824.1|       --------AIMRDI------------------------------------
R_gi|109476840|ref|XP_00106164      --------AIMRDI------------------------------------
M_gi|111607496|ref|NP_666257.2      --------AIMRDI------------------------------------
R_gi|2288925|emb|CAA04187.1|        --------AIMRDI------------------------------------
H_ENSP00000294423                   --------AIMRDI------------------------------------
H_ENSP00000264818                   --------TILRDL------------------------------------
M_gi|133922607|ref|NP_061263.2      --------TILRDL------------------------------------
M_gi|156630890|sp|Q62137.2|JAK      --------AILRDL------------------------------------
R_gi|2499671|sp|Q63272.1|JAK3_      --------AILRDL------------------------------------
H_ENSP00000222246                   --------AVIRDL------------------------------------
M_gi|114326478|ref|NP_00104164      --------AVIRDL------------------------------------
R_gi|2499669|sp|Q62689.1|JAK2_      --------AVIRDL------------------------------------
H_ENSP00000371067                   --------AIIRDL------------------------------------
D_gi|23093524|gb|AAN11824.1|AE      --------TIRTKL------------------------------------
D_gi|7294467|gb|AAF49811.1|         --------TIRTKL------------------------------------
D_gi|28380344|gb|AAF53079.3|        --------LVRMHL------------------------------------
H_gi|40254426|ref|NP_000897.2|      --------QIRLTL------------------------------------
R_gi|204270|gb|AAA41202.1|          --------QIRLAL------------------------------------
R_gi|477540|pir||A49183             --------QIRLAL------------------------------------
H_gi|4580422|ref|NP_003986.2|       --------QIKGFI------------------------------------
R_gi|16758694|ref|NP_446290.1|      --------QIKGFI------------------------------------
M_gi|20826487|ref|XP_131378.1|      --------QIKGFI------------------------------------
D_gi|23171402|gb|AAF55244.2|        --------TLKSMI------------------------------------
D_gi|7291217|gb|AAF46649.1|         --------TIRSNI------------------------------------
M_gi|159110415|ref|NP_032218.2      --------LTFDLFKSI---------------------------------
R_gi|13242283|ref|NP_077356.1|      --------LTFDLF------------------------------------
H_gi|4504217|ref|NP_000171.1|       --------HTFDLF------------------------------------
R_gi|18543337|ref|NP_570093.1|      --------QIYTQF------------------------------------
M_gi|28529710|ref|XP_142224.2|      --------EIFNQKLW----------------------------------
M_gi|20349229|ref|XP_111982.1|      --------EIFNQKLW----------------------------------
R_gi|16758684|ref|NP_446283.1|      --------EIFNQF------------------------------------
H_gi|134152694|ref|NP_001513.2      --------EIFNQF------------------------------------
H_gi|18386335|gb|AAB19934.2|        --------KIETTL------------------------------------
M_gi|21707860|gb|AAH34064.1|        --------KIESTL------------------------------------
H_gi|4758606|ref|NP_004508.1|       --------MIVPILEKM---------------------------------
R_gi|19173772|ref|NP_596900.1|      --------MIVPILEKM---------------------------------
D_gi|24667933|ref|NP_525001.2|      --------MVVPILEKM---------------------------------
H_gi|22749323|ref|NP_689862.1|      --------EILKKL------------------------------------
M_gi|12858445|dbj|BAB31320.1|       GRSLSGRERILERL------------------------------------
H_gi|6005792|ref|NP_009130.1|       --------EVLNTL------------------------------------
M_gi|28804246|emb|CAD29448.2|       --------EVLSSL------------------------------------
H_gi|57997202|emb|CAD38856.2|       -------LQDIRYI------------------------------------
M_gi|158635954|ref|NP_113563.2      -------LQDIRYI------------------------------------
D_gi|21358011|ref|NP_651655.1|      --------ELKQ-----IAYF-----------------------------
H_gi|7243101|dbj|BAA92598.1|        --------QMTK-----IPFF-----------------------------
H_gi|14041796|dbj|BAB55454.1|       --------RFLQ--------------------------------------
H_gi|115430241|ref|NP_065731.3      --------RFLQ--------------------------------------
M_gi|12963867|ref|NP_076401.1|      --------RFLQNCRAPGGFMSNRFVETNLFLEEIQIKEPAEKQKFFQEL
D_gi|7301824|gb|AAF56933.1|         --------DIITRCRKPGGFF-----------------------------
H_gi|15779207|gb|AAH14662.1|        --------TLLS-----HDFF-----------------------------
M_gi|20829352|ref|XP_129532.1|      --------TLLS-----HDFF-----------------------------
D_gi|45446806|gb|AAF45995.2|        --------ELLE-----HPIF-----------------------------
H_gi|18676872|dbj|BAB85045.1|       --------ELMK-----DKVF-----------------------------
H_gi|31982929|ref|NP_057524.2|      MLRN-NLEALLQDL------------------------------------
M_gi|21703091|gb|AAM74471.1|        TLRN-SLEALLQDM------------------------------------
H_gi|7020363|dbj|BAA91097.1|        PQFKIP-TKLKEALRIAKECIEKRLIEEQKQIHQHRRLTRAQSH------
M_gi|20869393|ref|XP_127567.1|      PQFKIP-TKLKEALRIAKECIEKRLTEEQKQIHQHRRL------------
D_gi|21627748|gb|AAM68879.1|        --------------------------------------------------
D_gi|22945875|gb|AAN10635.1|AE      --------------------------------------------------
D_gi|113194917|gb|AAF51744.3|       --------ELLESEFF----------------------------------
H_gi|58530886|ref|NP_001561.3|      Q-------EVCGSV------------------------------------
D_gi|21626698|gb|AAF47134.2|        T-------ALKHPWF-----------------------------------
D_gi|17368346|sp|P83097|WSCK_D      DV------------------------------------------------
D_gi|21626698_cp                    D-------CLEHRWL-----------------------------------
M_gi|12839087|dbj|BAB24429.1|       --------------------------------------------------
H_gi|13027388|ref|NP_061041.2|      --------SLLSHVFF----------------------------------
D_gi|17862032|gb|AAL39493.1|        --------KLMTHSFL----------------------------------
H_gi|16306492|ref|NP_203698.1|      -------MALNHPYF-----------------------------------
D_gi|24641273                       --------DIFSR-------------------------------------
H_gi|34191428|gb|AAH36504.2|        --------DVVHITFL----------------------------------
H_gi|17368698|sp|Q9BXU1|STK31_      ---------LNAECF-----------------------------------
M_gi|20895826|ref|XP_139682.1|      SP-----RDLSEEV------------------------------------
D_gi|21357711|ref|NP_647767.1|      MP------MIGARF------------------------------------
                                                                                      

D_gi|5052670|gb|AAD38665.1|AF1      ----
H_gi|14042287|dbj|BAB55185.1|       ----
H_gi|22760645|dbj|BAC11278.1|       ----
H_gi|12052916|emb|CAB66632.1|       ----
M_gi|21704242|ref|NP_663596.1|      ----
R_gi|13027458|ref|NP_076490.1|      ----
H_gi|22760572|dbj|BAC11248.1|       ----
H_gi|4758292|ref|NP_004436.1|       ----
M_gi|21594381|gb|AAH31924.1|        ----
H_gi|119534|sp|P21860.1|ERBB3_      ----
R_gi|149044565|gb|EDL97824.1|       ----
R_gi|109476840|ref|XP_00106164      ----
M_gi|111607496|ref|NP_666257.2      ----
R_gi|2288925|emb|CAA04187.1|        ----
H_ENSP00000294423                   ----
H_ENSP00000264818                   ----
M_gi|133922607|ref|NP_061263.2      ----
M_gi|156630890|sp|Q62137.2|JAK      ----
R_gi|2499671|sp|Q63272.1|JAK3_      ----
H_ENSP00000222246                   ----
M_gi|114326478|ref|NP_00104164      ----
R_gi|2499669|sp|Q62689.1|JAK2_      ----
H_ENSP00000371067                   ----
D_gi|23093524|gb|AAN11824.1|AE      ----
D_gi|7294467|gb|AAF49811.1|         ----
D_gi|28380344|gb|AAF53079.3|        ----
H_gi|40254426|ref|NP_000897.2|      ----
R_gi|204270|gb|AAA41202.1|          ----
R_gi|477540|pir||A49183             ----
H_gi|4580422|ref|NP_003986.2|       ----
R_gi|16758694|ref|NP_446290.1|      ----
M_gi|20826487|ref|XP_131378.1|      ----
D_gi|23171402|gb|AAF55244.2|        ----
D_gi|7291217|gb|AAF46649.1|         ----
M_gi|159110415|ref|NP_032218.2      ----
R_gi|13242283|ref|NP_077356.1|      ----
H_gi|4504217|ref|NP_000171.1|       ----
R_gi|18543337|ref|NP_570093.1|      ----
M_gi|28529710|ref|XP_142224.2|      ----
M_gi|20349229|ref|XP_111982.1|      ----
R_gi|16758684|ref|NP_446283.1|      ----
H_gi|134152694|ref|NP_001513.2      ----
H_gi|18386335|gb|AAB19934.2|        ----
M_gi|21707860|gb|AAH34064.1|        ----
H_gi|4758606|ref|NP_004508.1|       ----
R_gi|19173772|ref|NP_596900.1|      ----
D_gi|24667933|ref|NP_525001.2|      ----
H_gi|22749323|ref|NP_689862.1|      ----
M_gi|12858445|dbj|BAB31320.1|       ----
H_gi|6005792|ref|NP_009130.1|       ----
M_gi|28804246|emb|CAD29448.2|       ----
H_gi|57997202|emb|CAD38856.2|       ----
M_gi|158635954|ref|NP_113563.2      ----
D_gi|21358011|ref|NP_651655.1|      ----
H_gi|7243101|dbj|BAA92598.1|        ----
H_gi|14041796|dbj|BAB55454.1|       ----
H_gi|115430241|ref|NP_065731.3      ----
M_gi|12963867|ref|NP_076401.1|      SKSL
D_gi|7301824|gb|AAF56933.1|         ----
H_gi|15779207|gb|AAH14662.1|        ----
M_gi|20829352|ref|XP_129532.1|      ----
D_gi|45446806|gb|AAF45995.2|        ----
H_gi|18676872|dbj|BAB85045.1|       ----
H_gi|31982929|ref|NP_057524.2|      ----
M_gi|21703091|gb|AAM74471.1|        ----
H_gi|7020363|dbj|BAA91097.1|        ----
M_gi|20869393|ref|XP_127567.1|      ----
D_gi|21627748|gb|AAM68879.1|        ----
D_gi|22945875|gb|AAN10635.1|AE      ----
D_gi|113194917|gb|AAF51744.3|       ----
H_gi|58530886|ref|NP_001561.3|      ----
D_gi|21626698|gb|AAF47134.2|        ----
D_gi|17368346|sp|P83097|WSCK_D      ----
D_gi|21626698_cp                    ----
M_gi|12839087|dbj|BAB24429.1|       ----
H_gi|13027388|ref|NP_061041.2|      ----
D_gi|17862032|gb|AAL39493.1|        ----
H_gi|16306492|ref|NP_203698.1|      ----
D_gi|24641273                       ----
H_gi|34191428|gb|AAH36504.2|        ----
H_gi|17368698|sp|Q9BXU1|STK31_      ----
M_gi|20895826|ref|XP_139682.1|      ----
D_gi|21357711|ref|NP_647767.1|      ----
                                        
